# Temporal trend in dementia incidence since 2002 and projections for prevalence in England and Wales to 2040: modelling study

**DOI:** 10.1136/bmj.j2856

**Published:** 2017-07-05

**Authors:** Sara Ahmadi-Abhari, Maria Guzman-Castillo, Piotr Bandosz, Martin J Shipley, Graciela Muniz-Terrera, Archana Singh-Manoux, Mika Kivimäki, Andrew Steptoe, Simon Capewell, Martin O’Flaherty, Eric J Brunner

**Affiliations:** 1 Department of Epidemiology & Public Health, University College London WC1E 7HB, UK; 2 Department of Public Health and Policy, University of Liverpool, UK; 3 Department of Prevention and Medical Education, Medical University of Gdansk, Poland; 4 Centre for Dementia Prevention, University of Edinburgh, UK; 5 INSERM, U1018, Centre for Research in Epidemiology & Public Health, Hôpital Paul Brousse, France; 6 Clinicum, Faculty of Medicine, University of Helsinki, Finland

## Abstract

**Objective** To forecast dementia prevalence with a dynamic modelling approach that integrates calendar trends in dementia incidence with those for mortality and cardiovascular disease.

**Design** Modelling study.

**Setting** General adult population of England and Wales.

**Participants** The English Longitudinal Study of Ageing (ELSA) is a representative panel study with six waves of data across 2002-13. Men and women aged 50 or more years, selected randomly, and their cohabiting partners were recruited to the first wave of ELSA (2002-03). 11392 adults participated (response rate 67%). To maintain representativeness, refreshment participants were recruited to the study at subsequent waves. The total analytical sample constituted 17 906 people. Constant objective criteria based on cognitive and functional impairment were used to ascertain dementia cases at each wave.

**Main outcome measures** To estimate calendar trends in dementia incidence, correcting for bias due to loss to follow-up of study participants, a joint model of longitudinal and time-to-event data was fitted to ELSA data. To forecast future dementia prevalence, the probabilistic Markov model IMPACT-BAM (IMPACT-Better Ageing Model) was developed. IMPACT-BAM models transitions of the population aged 35 or more years through states of cardiovascular disease, cognitive and functional impairment, and dementia, to death. It enables prediction of dementia prevalence while accounting for the growing pool of susceptible people as a result of increased life expectancy and the competing effects due to changes in mortality, and incidence of cardiovascular disease.

**Results** In ELSA, dementia incidence was estimated at 14.3 per 1000 person years in men and 17.0/1000 person years in women aged 50 or more in 2010. Dementia incidence declined at a relative rate of 2.7% (95% confidence interval 2.4% to 2.9%) for each year during 2002-13. Using IMPACT-BAM, we estimated there were approximately 767 000 (95% uncertainty interval 735 000 to 797 000) people with dementia in England and Wales in 2016. Despite the decrease in incidence and age specific prevalence, the number of people with dementia is projected to increase to 872 000, 1 092 000, and 1 205 000 in 2020, 2030, and 2040, respectively. A sensitivity analysis without the incidence decline gave a much larger projected growth, of more than 1.9 million people with dementia in 2040.

**Conclusions** Age specific dementia incidence is declining. The number of people with dementia in England and Wales is likely to increase by 57% from 2016 to 2040. This increase is mainly driven by improved life expectancy.

## Introduction

It has been predicted that by 2050 well in excess of 100 million people worldwide will have dementia.^1^ Current costs of dementia to the UK economy are estimated at £23bn ($29bn; €26bn) annually.^2^ Burden of disability and years of life lost due to dementia in the UK increased by 76% between 1990 and 2010.^3^ Accurate projections for burden of dementia is a key step for planning to meet future needs.

Projections of dementia burden based on constant prevalence or incidence rates may not be precise,^4^ ^5^ ^6^ as they will only reflect population aging. Accurate predictions rely on accounting for changes in dementia incidence as well as changes in mortality rates. The competing effect of cardiovascular risk on future projections of dementia is also important. Alzheimer’s disease, vascular dementia, and cardiovascular disease share risk factors.^7^ ^8^ Thus, vascular risk reduction is likely to drive down age specific dementia incidence while, in contrast, leading to increased life expectancy and larger numbers susceptible to dementia.^9^ Given the opposing effects, simultaneous modelling of cardiovascular disease, dementia, and mortality is likely to enhance the accuracy of projections. Addressing shared determinants of disease follow recommendations from the National Institute for Health and Care Excellence,^10^ Public Health England, and the World Health Organization in the Blackfriars consensus.^11^ Such modelling approaches are, however, currently lacking.^4^

A key determinant of the future burden of dementia will be the underlying incidence trend. Although the balance of evidence suggests dementia incidence is declining,^12^ ^13^ ^14^ ^15^ ^16^ ^17^ ^18^ the magnitude of the decline is less certain. The ideal approach to determine time trends in dementia incidence would be based on continuous monitoring of a defined and representative population using a standard approach for case identification.^13^ ^19^ ^20^ In large epidemiological studies, changes in clinical criteria^21^ ^22^ and poor diagnostic agreement among clinicians^23^ are sources of variation in measured dementia incidence over time. Another challenge in establishing time trends is higher dropout among cohort study participants affected by, or in the preclinical stages of, dementia,^24^ ^25^ which leads to inaccurate estimates of dementia incidence and calendar trends.

In the present study, we developed a novel probabilistic Markov method (IMPACT-Better Ageing Model) to simultaneously model the transitions of the population through states of health, cardiovascular disease, cognitive and functional impairment, and dementia, to death, to obtain projections for the prevalence of dementia in England and Wales up to 2040. To account for the effect of selective dropout of study participants and to obtain unbiased estimates for dementia incidence over the past 15 years, with which to inform the model, we applied a robust joint modelling technique^26^ to data from the English Longitudinal Study of Ageing.

### Methods

#### Study population and sample

The English Longitudinal Study of Ageing (ELSA) sample was recruited in 2002-03 from participants of the 1998-2001 health surveys for England.^27^ ^28^ The sample was drawn by postcode sector, stratified by health authority and proportion of households in non-manual socioeconomic groups. A total of 12 099 men and women participated (response rate 67%), including 11 392 people aged 50 or more selected through the random sampling and 707 cohabiting partners. Survey weights were applied to ensure study participants formed a representative sample. Comparison of sociodemographic characteristics against national census indicated the ELSA sample was broadly representative of the population of England.^27^ To maintain representativeness of the study sample, refreshment participants were recruited to the study at wave 3 (2006-07; ages 50-55), wave 4 (2008-09; ages 50-74), and wave 6 (2012-13; ages 50-55) all drawn from the health surveys of England for the preceding years. A total of 17 906 participants were recruited to the study between waves 1 and 6 (see supplementary figure 1). Wave 7 data (2014-15) was not included in the analysis other than to identify transient impairments in cognition or function. At each wave, participants were interviewed to collect extensive demographic, medical, and lifestyle data. Clinical examinations were conducted at waves 2, 4, and 6. Participants provided written informed consent.

### Assessment of cognitive function in ELSA

Three sets of cognitive function tests were administered at every wave of ELSA. These tests, and method of being administered, include orientation to time, day, month, and year; immediate and delayed memory: one noun from a list of 10 is presented every two seconds to the participant who is then asked to recall as many words as possible immediately and after a short delay; and verbal fluency: participants are asked to name as many animals as possible in one minute. At waves 1, 4, and 6 a test of numeracy function was carried out by asking participants to solve four simple mathematics problems. At wave 6 an additional test of literacy was carried out by asking participants to deduce from a medicine label the number of days the medicine should be taken. Orientation to time was used to assess concentration, scores on the immediate and delayed recall were used as a measure of memory function, and scores on the animal naming, literacy, and numeracy test were used to measure verbal fluency and executive function.

The short version of the informant questionnaire for cognitive decline (IQCODE) was administered for participants unable to take part in the study who provided consent in advance or through a consultee.^29^ The IQCODE comprises 16 questions asking a proxy informant (usually an immediate family member) how the participant’s state of memory, ability to learn new tasks, judgment, and handling of key everyday situations (eg, making decisions on every day matters, or handling money for shopping) are compared with two years ago. The answers are graded on 5 point scales, from much improved to much worse. Use of the IQCODE questionnaire to identify cognitive impairment has been previously validated.^29^ The participant or proxy informants were asked about any doctor diagnosis of dementia.

### Assessment of functional impairment

Participants or proxy informants were asked about the ability of the participant to independently conduct basic activities of daily living. Such activities are key tasks related to self care and consist of getting in or out of bed, walking across a room, bathing or showering, using the toilet, dressing, cutting food, and eating. Impairment in independently performing one or more activities of daily living was defined as functional impairment. We considered impairment in conducting activities of daily living reported once, where the participant fully recovered at all further waves of data collection, to be transient and did not categorise those as functional impairment. Transient impairments at wave 6 were identified using wave 7 data.

### Case definition of cognitive impairment and dementia

We used an operational criteria based on cognitive function tests and IQCODE to define cognitive impairment.

*Cognitive function tests*—we used the criteria adapted for cognitive impairment no dementia (CIND).^19^ ^30^ Cognitive impairment was defined as impairment in two or more domains of cognitive function. Impairment in each domain of cognitive function is defined as a score of 1.5 standard deviations below the mean or lower compared with the population aged 50-80 years with the same level of education. Education was categorised in three levels: no qualification; O-level, A-level, or equivalent; and higher (university) education. We did not find considerable differences between men and women in the distribution of cognitive function scores after adjustment for age and education. We did not find evidence of large learning effects in consecutive cognitive function tests. The annual age specific and sex specific decline observed among participants who conducted the tests four or more years apart was similar to participants who conducted the tests two years apart. The cognitive assessment was considered invalid if the participant had responded to fewer than three tests on the cognitive battery. To avoid the effect of transient cognitive decline, resulting from delirium or other mental disorders, if the participant improved by 1 SD or more on cognitive tests at the consecutive wave, they were considered to not have cognitive impairment. Transient impairments of cognition at wave 6 were identified through cognitive assessment at wave 7.

*IQCODE*—a cut point of 3.3-3.6 is used for identification of cognitive impairment based on the IQCODE.^29^ We used a conservative cut-point of 3.6 for specificity.

Dementia caseness was defined either as a combination of cognitive impairment (according to the previously described definitions) and functional impairment (difficulty in performing one or more activities of daily living), or self reported doctor diagnosis of dementia. The operational definition adapted in this study conforms to criteria from the *Diagnostic and Statistical Manual of Mental Disorders*, fourth edition, for diagnosis of dementia (see supplementary file, section 1, for explanation).

### Cardiovascular disease and mortality

Cardiovascular disease in ELSA was ascertained by self reported doctor diagnosis of myocardial infarction, stroke, angina, coronary artery bypass grafting, or death from cardiovascular causes. Incidence of cardiovascular disease was defined as a first ever record of disease or intervention for each participant. Date and cause of death for ELSA study participants are obtained by data linkage with the UK Office for National Statistics.

### Statistical analysis

#### Trends in dementia incidence

We estimated the calendar trend in age specific dementia incidence across 2002-13 in ELSA in three statistical models with increasing complexity. Firstly, we estimated the calendar trend by fitting a Cox proportional hazards regression with incident dementia as the outcome and terms for age, age squared, sex, interactions of age and sex, and calendar time. Date of dementia was the mid-point between the wave in which dementia was reported or ascertained and the latest previous assessment. For participants who would only be classified as having dementia if they had impairment in numeracy function (assessed at waves 1, 4, and 6), the date of ascertainment was the mid-point between the two consecutive assessments that included the numeracy function test. The Cox proportional hazards analysis does not correct for bias as a result of selective dropout or the competing effect of mortality.

In the second stage, we fitted a competing risks model^31^ ^32^ with incident dementia as the outcome and mortality as a competing risk. Unlike a Cox proportional hazards model, in competing risks analysis, participants who die are not censored uninformatively and the effect of change in mortality over time on calendar trends in dementia incidence is accounted for.

At the third stage, to account for non-random dropout as well as competing risks of mortality, we fitted a joint model of longitudinal and time to event data.^26^ The longitudinal outcome of the joint model was the average standardised cognitive function test score, and the survival outcome was incident dementia. The survival outcome (incident dementia) was dependent on both the current value and the slope of the trajectory of the longitudinal outcome (standardised score on cognitive function tests). Other independent covariates in the model were age at entry, age squared, time since entry in the study (representing the effect of aging), time squared, sex, calendar year, level of education, and midlife history of obesity, hypertension, and diabetes. The longitudinal component allowed random intercepts and random slopes for the effect of age at entry and time since entry to the study. From the joint model we obtained individual level predictions for probability of dementia for ELSA study participants who were alive, including those lost to follow-up. To obtain calendar trends in incidence of dementia, we fitted linear regression models with log odds of incident dementia as the outcome to the data with terms for sex, age, age squared, interaction of sex with age and with age squared, and calendar time. The validity of this method to obtain predictions of incident dementia was assessed by comparing joint model predictions for future waves with data driven observed incidence rates. We validated the dementia case definition by comparing age specific incidence rates with those obtained from the Cognitive Function and Ageing Study-II.^13^

We examined the effect of changes in potential vascular and lifestyle risk factors on calendar trends in dementia incidence by entering risk factors as time varying covariates in regression models, with log odds of dementia as the outcome. The method for assessment of risk factors is shown in section 1 of the supplementary material.

#### Trends in dementia prevalence—IMPACT-BAM

To obtain valid projections for dementia prevalence to 2040, we developed IMPACT-BAM (fig 1), a probabilistic discrete time Markov model. IMPACT-BAM models transitions of the England and Wales population aged 35 or more years through states of illness and mortality. The model is initially populated using age-sex specific prevalence estimates, with transition probabilities applied at each one year iteration to predict number of deaths and prevalence of each of the eight states of IMPACT-BAM at the next calendar year. The model predicts future prevalence of cardiovascular disease, dementia, and functional impairment in addition to life expectancy, disabled and disability-free life expectancy, and mortality.

**Figure f1:**
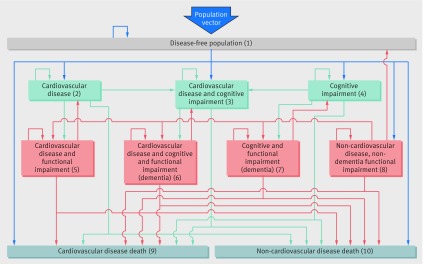
**Fig 1** IMPACT-Better Ageing Model (IMPACT-BAM). Numbers represent different health states and mortality. Population vector represents the number of men and women reaching age 35 and entering the model at each calendar year. States 6 and 7 represent dementia. States 5, 6, 7, and 8 represent functional impairment or disability

Input data to inform IMPACT-BAM include the population structure, the age-sex specific initial prevalence of each health state in the model, and age-sex-calendar time specific transition probabilities between states.

We obtained the numbers of the population in England and Wales in each stratum of age and sex at year 2006 (the start of the IMPACT-BAM model iterations) from ONS. Numbers of men and women who reach age 35 and enter the model at each calendar year are also obtained from ONS predictions. The entering cohort of 35 year olds is assumed to be free of cardiovascular disease and cognitive and functional impairment.

The baseline age and sex specific prevalence of each health state of the model was calculated using six waves of ELSA data pooled together and attributed to mid-point of the data collection timeframe, in 2006. The values obtained from this method corresponded to values observed at wave three (2006-08; see supplementary figure 2). We then used the curve fitting tool in MATLAB to obtain data for single year of age, starting at age 35 years.

The age-sex specific probability of transition from state*_i_* to state*_j_* in IMPACT-BAM (transition probability (TP*_ij_*)) for 2006 was obtained by fitting a logistic regression model on ELSA data with state*_j_* as outcome and terms for age, sex, interaction of age and sex, and a variable defining the initial state (state*_i_*). Transition probabilities to CIND (states 3 and 4 in figure 1) additionally included terms for age squared and its interaction with sex. We pooled transitions from wave n to wave n+1 in ELSA so that each individual contributed as many observations as corresponded to the number of two year epochs in which they participated in the study until being censored. We used a logistic model rather than a Cox proportional hazards model because the two years between data collection waves were relatively constant between participants and over time, and, unlike hazard ratios, odds ratios can be transformed into transition probabilities. Margins of the model provide two year transition probabilities for each stratum of sex, and single year of age at 2006. The two year probability (P) was then translated into one year transition probability using the formula: TP=1−exp((ln(1−P))/2)

All transition probabilities entered in IMPACT-BAM are calendar time specific. We calculated probabilities of cardiovascular and non-cardiovascular mortality up to 2040 in five year age bands using the Bayesian Age Period Cohort (BAPC) model with ONS mortality and population estimates from 1982-2012 for England and Wales as inputs (see supplementary figure 3).^33^ Mortality rates from each health state were assumed to change in parallel with overall cardiovascular and non-cardiovascular mortality rates. We calculated health state specific transition probabilities to cardiovascular and non-cardiovascular mortality by estimating the odds ratios of death from each health state using ELSA data (see supplementary table 1) and using the odds ratios to obtain the probability of deaths from cardiovascular and non-cardiovascular causes from each health state compared with that in the general population. To obtain state specific cardiovascular and non-cardiovascular mortality rates we applied the probability ratios to overall cardiovascular and non-cardiovascular mortality rates shown in supplementary figure 3. The decline in cardiovascular incidence paralleled the decline in cardiovascular mortality in ELSA (see supplementary figure 4). Hence we used the annual percentage change in cardiovascular mortality to estimate temporal change in cardiovascular incidence. The calendar trend for cognitive impairment or dementia was obtained from the joint model previously described. The effect of calendar time was imposed on the transition probabilities for 2006 to obtain transition probabilities for future years.

The IMPACT-BAM model was implemented in R statistical software and a package specifically written for it by author PB. Stata-14 (StataCorp 2015. College Station, TX: StataCorp LP) was used for data management and regression analysis to derive model inputs. The R package “JM” was used for joint modelling of longitudinal and time to event data.

#### Assumptions and sensitivity analysis

Table 1 presents the underlying assumptions for IMPACT-BAM and the evidence supporting the assumptions. We conducted sensitivity analyses to address uncertainties in these assumptions. We considered four alternative scenarios for calendar trend in dementia incidence: calendar trend obtained from analysis on ELSA data; a 2% relative annual decline as inferred by the Framingham Heart Study^18^; a 1.1% relative annual decline as inferred by Cognitive Function and Ageing Study-I and Cognitive Function and Ageing Study-II^13^; and no calendar decline in dementia incidence. We conducted a sensitivity analysis assuming the incidence of cardiovascular disease will no longer decline and will remain stable after 2014.

**Table 1 tbl1:** Summary of assumptions underlying the IMPACT-BAM model

Assumption	Justification
IMPACT-BAM models health transitions in the population of England and Wales aged 35 or more through to death. The input data for the probabilistic Markov model are the population size in each age and sex stratum, initial health state prevalence values, and transition probabilities by age, sex, and calendar year	
**Population numbers by age and sex**	
Estimates for population numbers by sex and five year age groups at model baseline were obtained from the UK Office for National Statistics (ONS). At each one calendar year iteration of the model, men and women reaching age 35 were entered. The predictions for number of people aged 35 by year were obtained from ONS Assumption 1: ONS predictions are realistic Assumption 2: migration is not a major source of bias	ONS provides official estimates for population demographics
**Starting prevalence values**	
Initial prevalence of health states in the model by age and sex were obtained from the English Longitudinal Study of Ageing (ELSA) Assumption 3: ELSA is a representative sample of the population of England and Wales	Accuracy of prevalence values depends on how well ELSA represents the population of England and Wales. ELSA study participants aged 50 or more were selected at random. The core participant’s cohabiting partners, including adults aged less than 50, were also enrolled in the study. The overall response rate was 67%. To ensure study participants form a representative sample, survey weights are applied. To maintain representativeness at every phase of data collection, refreshment samples are recruited to the study periodically. Comparisons of the sociodemographic characteristics of participants against results from the national census indicated that the ELSA sample was broadly representative of the English population
To improve statistical power, six waves of ELSA data were pooled. Prevalence estimates of cardiovascular disease and cognitive and functional impairment that define the health states were obtained from pooled data and attributed to 2006, which is the mid-point of the ELSA data collection timeframe and the baseline of the model Assumption 4: Prevalence estimates from six pooled waves of data provide a precise and accurate estimate of prevalence at mid-point of the data collection timeframe	The prevalence values obtained from the pooled ELSA data matched the prevalence values obtained at the mid-point (2006, wave 3). Estimates for prevalence of cardiovascular disease are displayed as an example in supplementary figure 2
Assumption 5: The prevalence of each health state at each calendar year from the starting point (2006) onwards, equals the number of people who were in that health state in the previous year, plus new incident cases, minus those who made the transition to another health state or died from any cause. Number of new incident cases and numbers of death were determined by transition probabilities to and from that condition	Epidemiological concept applied to Markov models
**Transition probabilities**	
Transition probabilities were obtained as a function of age and sex from incident cases between wave n and n+1 in ELSA. As with estimates of prevalence values, the transition probabilities obtained from pooling ELSA epochs were attributed to the mid-point of the data collection period Assumption 6: Transition probabilities, (equivalent to incidence by age, sex, or calendar year) for cardiovascular disease, dementia, functional impairment, and mortality in ELSA are similar to those for England and Wales	Incidence of cardiovascular disease and dementia by age and sex were consistent with age, and sex specific incidence values obtained from independent external sources for the corresponding calendar time Estimates of dementia incidence were available from the Cognitive Function and Ageing Study II (CFAS II, 2008-11). Incidence of dementia in the corresponding timeframe is compatible with CFAS-II estimates (see supplementary figure 5). Deaths predicted by IMPACT-BAM matched with observed and predicted mortality rates from the ONS (see supplementary figure 13)
Assumption 7: Transition probabilities are equivalent to a weighted average across the spectrum of the severity of each condition, thus varying severities among people in each health state is accounted for. Similarly, survival of those with each condition is assumed to be equivalent to the weighted average of survival of people with different levels of severity	Modelling is based on a single transition probability for each age, sex, and calendar year stratum and health transition. The probability of death or development of functional impairment among those with cardiovascular disease or cognitive impairment is dependent on the severity of cardiovascular disease or cognitive impairment. Under the assumption that ELSA participants are a representative sample of the population, the spectrum of the severity of conditions (eg, cardiovascular disease, or cognitive impairment) observed in ELSA is proportionate to that at population level. As such, transition probabilities obtained from ELSA are a weighted average of the transition probabilities across the spectrum of the severity of the conditions The weighted average transition probability multiplied by the total number of people in a health state is mathematically equivalent to the sum of the product of severity specific transition probabilities and severity specific numbers of people in that health state
Assumption 8: The effects of comorbidity (such as diabetes) are accounted for in the model	Since ELSA participants are assumed to be a representative sample of the population of England after weighting (see above), estimates for risks of dementia, cardiovascular disease, functional impairment and death obtained from ELSA reasonably represent a weighted average of risk levels across the spectrum of the severity of these conditions and comorbidities
**Calendar trends** Transition probabilities (mortality rates and incidence of cardiovascular disease and dementia) change over time	
Assumption 9: The observed downward calendar trend in mortality rates over the past two decades will continue to the future	Data obtained from ONS show that cardiovascular and non-cardiovascular mortality rates followed steady and linear downward trends over the past two decades. We assumed the most likely scenario would be that these trends will continue (see supplementary figure 3)
Assumption 10: Life expectancy and maximum lifespan are amenable to being increased	Changes in life expectancy are accounted for by application of mortality rates. As mortality rates continue to decline, life expectancy will increase
Assumption 11: Trends in incidence of cardiovascular disease over time are parallel to cardiovascular mortality	Age and sex standardised cardiovascular incidence and mortality rates declined in parallel in ELSA (see supplementary figure 4). To examine the uncertainty of this assumption, we conducted a sensitivity analysis assuming incidence of cardiovascular disease does not decline any further (results shown in supplementary figure 16)
Assumption 12: Dementia incidence declines over time	A decline in dementia incidence has been reported in studies in England, the Netherlands, and USA. The magnitude of the calendar trend in England and Wales is less certain. We determined the calendar trend corrected for deaths and loss to follow-up of study participants, utilising a robust statistical technique to model ELSA data We conducted sensitivity analyses with calendar trends estimated from other studies (including CFAS I and II, and the Framingham study), and an alternative scenario in which dementia incidence does not decline any further (fig 6)
Assumption 13: Survival with cardiovascular disease, dementia, or functional impairment change over time in parallel to changes in overall life expectancy	Survival in IMPACT-BAM is indirectly modelled as a function of changing mortality rates. It is assumed that the ratio of mortality rates for each health state in the model compared with the general population is similar to that observed in ELSA. Thus mortality and survival for each health state in the model changes in parallel to mortality and survival in the general population. Current evidence suggests survival with cardiovascular disease and dementia is improving over time. We did not find evidence suggesting this improvement to be over and beyond improvement in overall survival
Assumption 14: The most likely net effect of future changes in risk factors will be the continuation of calendar trends in mortality rates and incidence of dementia and cardiovascular disease observed over the past two decades	Population levels of risk factors affecting incidence of cardiovascular disease and dementia such as diabetes, smoking, diet, and physical activity have changed over time. The net effect of recent changes in risk factors on changes in mortality rates and incidence of cardiovascular disease and dementia has been steady and linear declining calendar trends The present analysis forms the baseline modelling scenario. IMPACT-BAM will be utilised in future to model the health impacts of changes in risk factors and public health interventions compared with the baseline scenario. Results of such analysis are extensive and beyond the scope of this paper
**Competing risks**	
Assumption 15: Deaths due to any cause (including cancer and chronic obstructive pulmonary disease) and changes in cause specific mortality rates act as competing risks to development of dementia	Cardiovascular and non-cardiovascular causes of death are the terminal health states in the model. Once a person dies from any cause they are no longer at risk of disease. Thus, competing risks due to both cardiovascular and non-cardiovascular causes are accounted for in the model

To explore the impact of parameter uncertainty on model outputs, we conducted a probabilistic sensitivity analysis using Monte Carlo simulation. The procedure entails sampling from specified distributions for the input parameters that were used in the model for each data cycle. We calculated 1000 iterations to estimate 95% uncertainty intervals for output variables.

#### Validation

To validate methods, definitions, and assumptions, we ran the model starting in 2006 to predict prevalence of dementia in 2011. We compared model estimates with the prevalence of dementia observed from the Cognitive Function and Ageing Study-II.^20^ Similarly, we compared the prevalence of cardiovascular disease with the health survey for England 2011,^34^ and mortality rates with data from the ONS. We also compared model predictions with dementia prevalence at wave 7 of ELSA.

### Patient involvement

Study participants were not involved in setting the research question or the outcome measures, nor were they involved in developing plans for design or implementation of the study. No participants were asked to advise on interpretation or writing up of results. There are no direct plans to disseminate the results of the research to study participants.

### Results

#### Trends in dementia incidence: ELSA (2002-13)

Supplementary table 2 shows the baseline characteristics of ELSA participants. Between 2002 and 2013 dementia was ascertained for a total of 1448 ELSA participants: 634 participants had dementia at the time of recruitment, and 814 incident cases were identified over the course of follow-up; 16.5% occurred in participants aged less than 65 years. Of the 1448 participants with dementia, 466 received a doctor diagnosis of dementia, 245 were identified through IQCODE questionnaire plus functional impairment in activities of daily living, and 1078 were identified by impairment in cognitive tests and impairment in one or more activities of daily living. Of the 245 participants with dementia based on IQCODE and functional impairment, 171 (70%) reported a doctor diagnosis of dementia. Among those with impairment in cognitive tests and activities of daily living function, 263 (25%) reported a doctor diagnosis of dementia at later stages or were considered to have dementia based on the IQCODE questionnaire.

Supplementary table 3 shows the number of incident dementia cases ascertained at each wave of data collection. Age-sex specific dementia incidence observed in ELSA was consistent with that observed in the independent Cognitive Function and Ageing Study-II study, whereas incidence rates corrected for the effect of dropout were higher (see supplementary figure 5). Incidence rates of dementia corrected for the effect of dropout were higher at older ages, marginally higher in women than men (see supplementary figures 6 and 7.A), and higher than uncorrected crude observed rates (see supplementary figure 7.B). In 2015, age standardised incidence of dementia in the population of England and Wales aged 50 or more years was estimated at 1.3% in men and 1.5% in women, corresponding to 125 800 new cases of dementia in men and 162 650 new cases in women.

The magnitude of the calendar trend in age specific dementia incidence, without correcting for either mortality or selective dropout, was obtained from Cox proportional hazards analysis based on participants who attended and remained in the study at each two year interval. The age adjusted and sex adjusted dementia incidence relatively decreased by 1.5% each year (hazard ratio 0.985, 95% confidence interval 0.954 to 1.018; see supplementary figure 8.A). Accounting for the competing risk of mortality in separate competing risks analysis yielded a steeper trend of −2.7% (0.973, 0.932 to 1.016). The effect of non-random dropout as well as changing mortality rates was accounted for by fitting joint models of longitudinal and time-to-event data. The corrected calendar trend in dementia incidence was a statistically significant relative reduction of 2.7% each year (odds ratio 0.973, 95% confidence interval 0.971 to 0.976; see supplementary figures 6 and 8.B). The relative annual reduction tended to be steeper in women (0.972, 0.968 to 0.976) than in men (0.975, 0.971 to 0.980), but the interaction by sex was not statistically significant. Changes over time in available risk factors accounted for about 25% of the calendar effect in dementia incidence (fully adjusted odds ratio 0.98 (95% confidence interval 0.977 to 0.982); see supplementary table 4).

#### Trends in dementia prevalence: IMPACT-BAM

Age-sex specific prevalence of dementia at each data collection wave of ELSA provides the starting values for the IMPACT-BAM model (see supplementary table 5). In ELSA, age-sex standardised prevalence of dementia declined over time (see supplementary figure 9). For the purpose of validating the model, IMPACT-BAM was populated with age-sex specific prevalence estimates and transition probabilities for 2006 to estimate prevalence of dementia, cardiovascular disease, and mortality in 2011. The age-sex specific dementia prevalences predicted by IMPACT-BAM for 2011 (see supplementary figure 10) were compatible with estimates from the independent Cognitive Function and Ageing Study-II. Model predictions also matched prevalence of dementia at wave 7 of ELSA (see supplementary figure 11). Cardiovascular disease prevalence and mortality rates were compatible with observations from independent sources (see supplementary figures 12 and 13).

In the analyses on future prevalence of dementia up to 2040 assuming a 2.7% relative annual decline in dementia incidence, the number of people with dementia in England and Wales is set to increase from 766 600 (95% uncertainty interval 735 200 to 796 900) in 2016 to 871 700 (835 600 to 906 600) in 2020, 1 091 600 (1 034 200 to 1 146 200) in 2030, and 1 204 500 (1 101 000 to 1 296 200) in 2040 (fig 2). Much of the increase in number of people with dementia occurs in the older age groups (fig 3, see supplementary figure 14). Overall crude prevalence of dementia in the population aged 50 or more is estimated at 3.5% (95% uncertainty interval 3.4% to 3.7%) in 2016, 3.8% (3.7% to 4.0%) in 2020, 4.3% (4.1% to 4.5%) in 2030, and 4.4% (4.0% to 4.7%) in 2040 (fig 4). The crude prevalence of dementia in population aged 50 or more and 65 or more is estimated to increase up to 2040 in men, whereas in women it declines after 2025 (fig 4). These crude prevalence estimates are affected by the population structure. The prevalence of dementia age standardised to the population of 2015 is estimated to decline by about 21% from 2016 to 2040 (fig 5).

**Figure f2:**
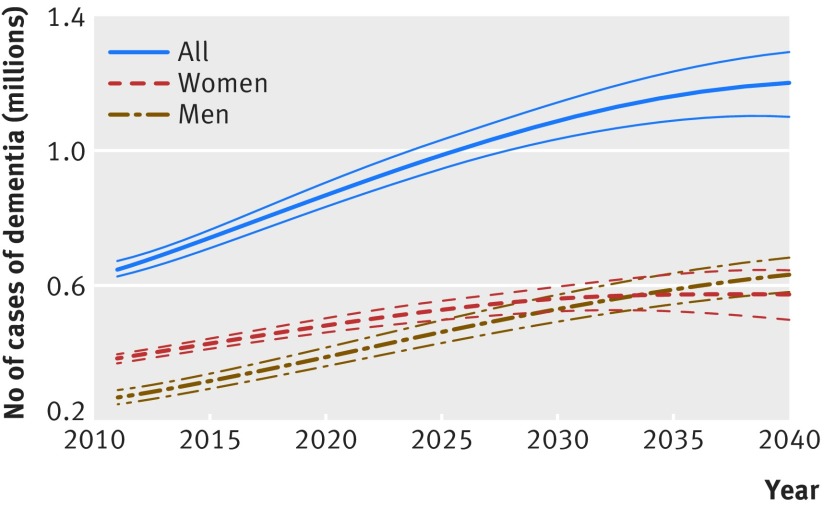
**Fig 2** Projected number of people with dementia in England and Wales 2011-40. Thinner lines represent 95% uncertainty intervals

**Figure f3:**
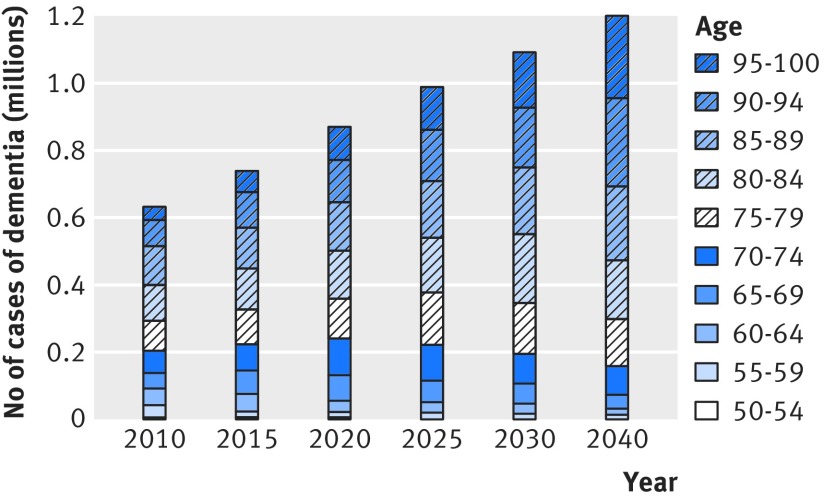
**Fig 3** Age specific estimated number of cases of dementia 2010-40 in men and women

**Figure f4:**
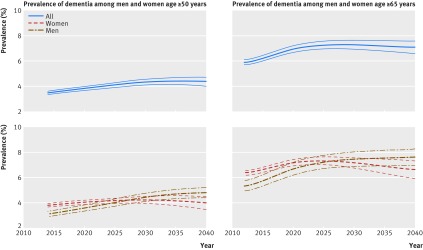
**Fig 4** Projected prevalence of dementia in England and Wales, 2011-40. Thinner lines represent 95% uncertainty intervals

**Figure f5:**
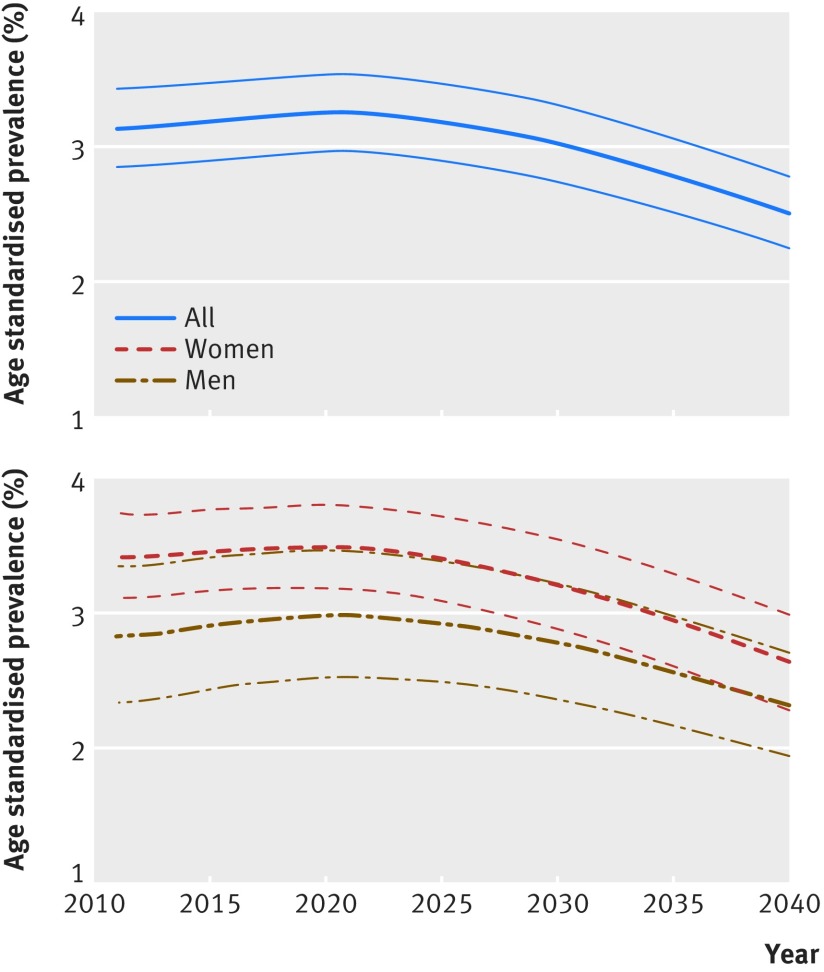
**Fig 5** Projected prevalence of dementia in England and Wales, 2011-40, age standardised to the population of 2015. Thinner lines represent 95% uncertainty intervals

Figure 6 shows the results of sensitivity analyses for predicted numbers of dementia cases based on different values for calendar trend in dementia incidence. Assuming no calendar trend in dementia incidence, the number of people with dementia is estimated at 1.9 million (95% uncertainty interval 1.76 million to 2.0 million) in 2040, with an increase rather than a decrease in age standardised prevalence of dementia (see supplementary figure 15). The predicted number of people with dementia under the assumption that cardiovascular disease incidence does not decline any further was 758 700 (95% uncertainty interval 728 200 to 788 000) in 2016 and 1 112 700 (1 023 400 to 1 192 700) in 2040 (see supplementary figure 16), 8% fewer compared with the scenario in which the incidence of cardiovascular disease continues to decline.

**Figure f6:**
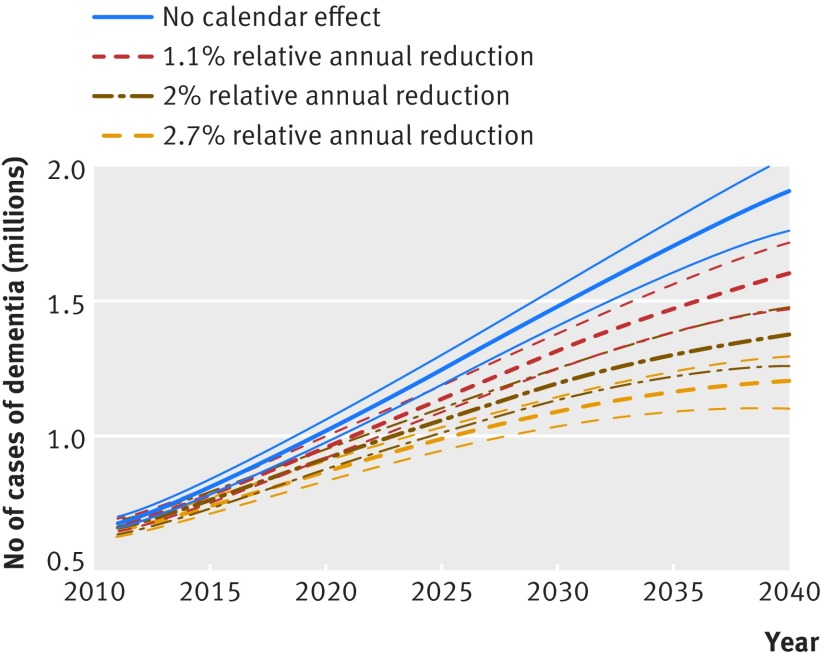
**Fig 6** Sensitivity analysis for number of cases of dementia under alternative assumptions for calendar trend in incidence of dementia. Thinner lines represent 95% uncertainty intervals

## Discussion

Our results shift the balance of evidence towards more certainty that dementia incidence is indeed decreasing. The decline, estimated at a relative reduction of 2.7% each year, was evident after accounting for mortality and non-random dropout from the study. Although age specific incidence of dementia is declining, the overall disease burden is set to increase substantially owing to increased life expectancy and declining rates of mortality and cardiovascular disease incidence. With current population projections, we estimate there will be a 57% increase in the number of people with dementia between 2016 and 2040, with more than 1.2 million people with dementia in England and Wales by 2040.

### Strengths and limitations of this study

To our knowledge, this is the first study to predict number of cases and prevalence of dementia in a population using methods that simultaneously model the observed trends in mortality, cardiovascular disease, and dementia. Previous predictions were based on constant age specific incidence^6^ or prevalence^2^ of dementia. An accurate projection of the number of people with dementia is only possible with a modelling strategy that accounts for the opposing effects of increasing life expectancy and declining dementia incidence, a requirement highlighted by the non-linearity of the generated estimates of prevalence in the present study. On the basis of the calendar effect derived from the English Longitudinal Study of Ageing (ELSA), there is an upward trend in numbers of people with dementia, but to a smaller degree than previously estimated. Previous forecasts of larger increases in dementia burden are based on less complex approaches that do not account for changes in dementia incidence, survival, or competing risks.^4^ ^35^ ^36^ ^37^ Under the scenario of assuming that the incidence of dementia does not decline, prevalence projections for dementia were higher, and were similar to those of Alzheimer’s Society UK.^2^

Changes in mortality rates are important determinants of the numbers of people with dementia as they govern both life expectancy and the pool of individuals susceptible to dementia, as well as survival in those affected by the condition,^5^ and thus are meticulously incorporated in the model. Cardiovascular and non-cardiovascular mortality rates have shown steady and linear downward trends in the past decades, and we assumed this trend is likely to continue. IMPACT-BAM shows that the decline in age standardised dementia prevalence, corresponding to the decline in incidence, is outweighed by population aging in the near future, and numbers of people with dementia are likely to increase rapidly between 2015 and 2030. In the following decade, however, the number of people with dementia will level out. Furthermore, the numbers of men and women with dementia is set to converge within the next 15 years. This finding can partly be explained by a faster decline in mortality rates in men compared with women (see supplementary figure 3) and narrowing of the life expectancy gap between the sexes.^9^

We derived the required inputs for IMPACT-BAM from best available data. ELSA is a large, representative sample of the population aged 50 years or more and surveyed using standard questions at two year intervals. Six waves of data allowed us to account for mortality and dropout from the study using robust statistical methods. Cognitive decline starts at a younger age^38^ than the 65 or more or 70 or more age groups recruited in previous studies.^12^ ^13^ ^18^ ^24^ ^39^ ^40^ ^41^ We attempted to fill the gap by capturing cognitive impairment and dementia starting at age 50. Model outputs were validated against observations from independent sources.

ELSA participants were not clinically screened for dementia, rather, an operational case definition based on standardised assessments of cognition and function was applied. The standardised assessment is comparable across time, and thus more informative of dementia trends than clinical assessments, which are likely to be affected by changes in diagnostic criteria^21^ ^22^ and clinical practice over time.^23^ The case definition applied in this study, follows DSM-IV and other clinical criteria (National Institute of Neurological Disorders and Stroke and the Association Internationale pour la Recherche et l’Enseignement en Neurosciences (NINDS-AIREN) and National Institute of Neurological and Communicative Disorders and Stroke and the Alzheimer’s Disease and Related Disorders Association (NINCDS-ADRDA)) in that it hinges on non-transient impairment in two or more cognitive domains, resulting in functional impairment (see supplementary file, section 1). Cognitive assessment in ELSA is based on a set of standard and validated^30^ ^42^ ^43^ ^44^ ^45^ cognitive function tests; none the less the list is not comprehensive. Cognitive impairment in domains other than those tested may have been missed, leading to underestimation of dementia cases. Similarity of age specific estimates of dementia incidence and prevalence with those of CFAS-II suggests this source of bias is small.

The case definition for dementia used in this study required moderate to severe impairments in cognition and function to minimise false positives. This results in inclusion of moderate to severe, rather than mild, cases of dementia. The main aim of this exercise is to inform future societal and healthcare needs. To this end, the dementia case definition is relevant to health and social policy as it forecasts numbers of people who would require supportive care owing to moderate or severe cognitive and functional impairment. Although residents of care homes were not included in ELSA, we took account of this group using the statistical joint modelling approach. Furthermore, data from carers and self reported doctor diagnosis of dementia identified cases among those unable to take part in the study. Accounting for non-participation and dropout from the study increased the obtained incidence rates for dementia, but not by a considerable amount (see supplementary figure 7), a finding consistent with the Mayo Clinic Study of Ageing.^24^

Survival with cardiovascular disease and dementia has improved over time.^5^ ^46^ ^47^ Changes in life expectancy and survival with cardiovascular disease, functional impairment, or dementia are accounted for in the model by applying calendar time specific mortality rates. This method is based on the assumption that survival with health conditions changes in proportion to changes in overall life expectancy. This assumption, although reasonable and commonly applied in modelling studies,^3^ ^6^ ^48^ lacks verification, as evidence on the exact age and sex specific survival with dementia is rare,^5^ and it is hard to obtain given uncertainty over date of onset.

### Comparison with other studies

Age specific and sex specific incidence rates of dementia obtained using the described methods (before correction for dropouts) were in line with age-sex specific incidence rates obtained in other European studies, including the population of England (CFAS-II,^13^ see supplementary figure 3), Italy (Italian Longitudinal Study of Ageing),^39^ and Spain (NEDICES study),^40^ higher than that in the Rotterdam study^12^ and relatively lower than American populations of Minnesota (Mayo Clinic Study of Ageing)^24^ and white participants of the Cardiovascular Health Study (1989-99).^41^ Although not suitable in a clinical setting, the similarity of our estimates for age specific dementia incidence with those of independent studies underpins the validity of our case definition for dementia at population level.

Several cohorts and regionally representative panel studies have reported calendar trends in dementia incidence.^13^ ^49^ Comparing findings of CFAS I and II studies^13^ showed a 20% decline in dementia incidence over 20 years, statistically significant only in men, using algorithmic diagnosis among participants who attended reassessment interviews within two years of CFAS-I (1989-94) and CFAS-II (2008-11). Our results, based, in parallel, on participants who remained in the study at biennial waves of ELSA also translate to a 20% decline in dementia incidence over 20 years. After accounting for the competing effect of mortality and dropouts, the annual reduction was, as expected, larger (2.7%). This corresponds to a 42% decline in dementia incidence over two decades, and it is statistically significant for both men and women. The corrected dementia trend, corresponding to a 24% decline for each decade, is consistent with findings from the Framingham study (20% decline for each decade across 1977-2008),^18^ the Rotterdam study (non-statistically significant 25% lower incidence in the 2000 compared with the 1990 subcohort),^12^ and the Chicago Health and Aging project (non-significant 3% annual reduction across 1997-2008).^14^ Other studies suggested a decline in dementia incidence, indirectly inferred from comparing prevalence estimates in Spain^50^ and Sweden.^15^ Some studies in the United States,^14^ China,^51^ and Japan^52^ found no statistically significant trend. No published study has reported evidence of an increasing trend in dementia incidence.^49^

Several plausible explanations support a decline in dementia incidence over time. Improvement in vascular risk factors,^7^ ^8^ ^53^ as well as in education levels, can partly account for the decline in incidence. In the present study, increased physical activity accounted for the largest proportion of the decline in dementia incidence between 2002 and 2013. Changes in prevalence of diabetes, smoking, and social class over time had negative confounding effects, such that the downward incidence trend increased after controlling for respective changes. Adjustment for stroke and depression did not have a considerable effect on the calendar trend in dementia.

### Policy implications

Basic research efforts to understand the causes of dementia have increased noticeably in recent years, but to date drug trials have failed to show modification of disease processes.^54^ The WHO and other expert bodies have identified prevention, identification, and reduction of risk as a top research priority, in response to the lack of treatment.^55^ ^56^ Debate continues about the relative importance of vascular and neurodegenerative causes. In the context of this uncertainty the IMPACT-BAM model is a means to understand how the dementia burden will evolve, and it provides a platform to measure how the burden might be reduced through various policy interventions.

### Conclusion

Our novel prediction model integrates recent downward trends in dementia and cardiovascular disease incidence with declining mortality rates in England and Wales. If these trends continue then the number of people with dementia will more than likely increase, from 792 000 in 2017 to more than 1.2 million in 2040. The projected increase in the burden of dementia, despite the substantial downward trend in age specific incidence, results largely from improvements in life expectancy. The results have important policy implications in terms of care needs and public spending. The findings of our prediction model act as a benchmark to measure the impact of possible dementia prevention initiatives.

**What is already known on this topic**

Alzheimer’s Society UK predicts if age specific prevalence of dementia remains constant, there will be over 1.7 million people with dementia in the UK by 2050
Studies in England, the Netherlands, and United States have shown a decline in dementia incidence over time

**What this study adds**

The decline in dementia incidence each year in 2002-13 was steeper than that observed in previous studies
The Markov model shows that despite a decline in age specific dementia incidence, overall prevalence of this condition is rising; however, the increase is not as large as predicted by simple projections of prevalence into the future
This study estimates that by 2040 in England and Wales there will be more than 1.2 million people with dementia, an increase of 57% from 2016

## Web extra

Extra material supplied by authors

## Notes

We thank all ELSA study participants and the study team for their contribution, Panayotes Demakakos for help with use of the ELSA dataset, and Dimitris Rizopoulos for statistical help.

Contributors: All authors made substantial contribution to the conception and design of this study. EJB and MOF developed the original idea for IMPACT-BAM. SAA, MJS, MGC, and PB analysed and prepared the input data for the model. PB and MGC developed and implemented the model, with input from MOF, SAA, MJS, EJB, and SC. SAA ran the joint model of longitudinal and time to event data, with input from MJS and GMT. SAA wrote the manuscript with coauthors. All authors contributed to interpreting the results, drafting the manuscript, and the revisions. SAA and EJB are the guarantors.

Ethical approval: This study was approved by the multicenter research and ethics committee.

Data sharing: Technical appendix with details on the formula and calculations for the IMPACT-BAM model is available from the corresponding author.

Transparency: The lead author (SAA) affirms that the manuscript is an honest, accurate, and transparent account of the study being reported; that no important aspects of the study have been omitted; and that any discrepancies from the study as planned have been explained.
