# Supplementary material for: Temporal trend in dementia incidence since 2002 and projections for prevalence in England and Wales to 2040: modelling study
Source: BMJ. 2017 Jul 5;358:j2856. doi: 10.1136/bmj.j2856 (PMC5497174; doi:10.1136/bmj.j2856)
Supplement: Supplementary file 1 — Supplementary information: additional material [file ahms034697.ww1.pdf]

Supplement Material

Temporal trend in dementia incidence since 2002 and projections for prevalence in England and Wales to 2040: a modelling study

Authors: **Sara Ahmadi-Abhari, Maria Guzman Castillo, Piotr Bandosz, Martin J Shipley, Graciella Muniz-Terrera, Archana Singh-Manoux, Mika Kivimäki, Andrew Steptoe, Simon Capewell, Martin O'Flaherty, Eric J Brunner**

Table of Contents:

Section 1: Supplement Methods ……………………………….……………………..… Page 3

1.1: Case definition of dementia in relation to DSM-IV criteria ………………………. Page 3

1.2: Assessment of Covariates in risk factor analysis ……………………..………… Page 4

Section 2: Supplement Results …………………………………………………………. Page 5

Supplement Figure 1: Flow-diagram of participants recruited to the English Longitudinal Study of Ageing (2002-2013) …...…………….………………………… Page 5

Supplement Figure 2: The prevalence of cardiovascular disease by age and sex obtained from pooling 6 waves of ELSA data (2002-2013), compared to prevalence estimates obtained at the mid-point of the time-frame (wave 3; 2006-2007)…………… Page 6

Supplement Figure 3: Observed (1993-2013) and projected (2014-2040) cardiovascular and non-cardiovascular mortality rates by age and sex. The mortality rates are displayed on a logarithmic scale……………………………………………………….. Page 7

Supplement Table 1: Odds ratio of cardiovascular and non-cardiovascular mortality for each health state in the IMPACT-BAM model obtained from the English Longitudinal Study of Ageing 2002-2013 ………………………………………………………………. Page 8

Supplement Figure 4: Age and sex standardised cardiovascular incidence and mortality rates in the English Longitudinal Study of Ageing 2002-2013 ……………………...... Page 9

Supplement Table 2: Baseline Characteristics of the English Longitudinal Study of Ageing participants ………………………………………………………………… Page 10

Supplement Table 3: Number of Incident cases of dementia at each wave of the English Longitudinal Study of Ageing (2002 – 2013) ………………………………… Page 11

Supplement Figure 5: Comparison of incidence (per 1000 person years) of dementia in English Longitudinal Study of Ageing (waves 4 to 6 (2008 – 2013)) with Cognitive Function and Ageing Study II (2008 – 2011) ………………………………………….. Page 12

Table of Contents Continued:

Supplement Figure 6: Age-sex specific trends in incidence of dementia – for years 2005, 2010, 2015 corrected for dropouts using joint models in the English Longitudinal Study of Ageing …………………………………………………………………………….. Page 13

Supplement Figure 7: Incidence of dementia as observed and corrected for dropouts using joint models, at mid-point of ELSA data collection period (2002-2013) ...……… Page 14

Supplement Figure 8: Incidence of dementia A) as observed and B) corrected for dropouts using joint models, for years 2005 and 2010 in men and women …….……... Page 15

Supplement Table 4: Calendar trend in dementia incidence adjusted for change in level of risk factors …..…………………………………………………………………... Page 16

Supplement Table 5: Observed prevalence (%) of dementia (95% confidence interval) by age-group at each wave of data collection for English Longitudinal Study of Ageing (ELSA) 2002-2013….……………………………………………………………… Page 17

Supplement Figure 9: Age- and sex-standardised prevalence (%) of dementia at each data collection wave of the English Longitudinal Study of Ageing (ELSA) 2002-2013 Page 18

Supplement Figure 10: IMPACT-BAM predicted estimates for prevalence of dementia compared with estimates from the Cognitive Function and Ageing Study (CFAS II) in 2011 ……………………………………………………………………………………….. Page 19

Supplement Figure 11: IMPACT-BAM predicted prevalence of dementia in men and women compared to estimates from wave 7 of the English Longitudinal Study of Ageing (2014-2015) ………………………………………………………………… Page 20

Supplement Figure 12: IMPACT-BAM predicted cardiovascular disease prevalence compared with observed estimates from the Health Survey for England in 2011 ……... Page 21

Supplement Figure 13: IMPACT-BAM predicted mortality compared with observed estimates from the UK Office for National Statistics ….………………………………. Page 22

Supplement Figure 14: Age and sex specific estimated number of cases of dementia 2010-2040 …….…………………………………………..…...……………….…… Page 23

Supplement Figure 15: Sensitivity analysis for prevalence of dementia assuming no calendar trend in incidence of dementia, standardized to the population of England and Wales in 2015 ….…………………………………………..…...……………….…… Page 24

Supplement Figure 16: Sensitivity analysis for numbers of cases of dementia assuming cardiovascular incidence does not decline after 2014 ………………………………….. Page 25

Reference list ….……………………………………...……………………………..… Page 26

Section 1: Supplement Methods:

Case definition of dementia in relation to DSM-IV criteria

We adapted the dementia case definition to resemble DSM-IV and other criteria (such as NINDES-AIREN and NINCDS-ADRDA) for diagnosis of dementia. The cornerstone of clinical diagnostic criteria for dementia is impairments in two or more cognitive domains that result in considerable loss of function. Thus, we defined cognitive impairment as a score of equal to or lower than 1.5 standard deviations below mean, standardized to the population aged 50-80 with the same level of education, similar to criteria used for defining cognitive impairment no dementia (CIND).[1] Loss of function was defined as impairments in conducting activities of daily living. We sought with a set of criteria to encompass all types of dementia and not merely Alzheimer's disease. Although memory impairment is a key element in the diagnosis of Alzheimer's disease, memory is affected to varying degrees in vascular, fronto-temporal and Lewy body types of dementia. Thus, memory impairment was not included as a necessary criterion in defining cognitive impairment in this study.

DSM-IV criteria specify that the disturbances do not occur exclusively during the course of delirium and are not better accounted for by another mental disorder. For the criteria to hold, and to increase specificity, transient impairments in cognitive function or conducting ADLs, were not classified as cognitive or functional impairment. Inclusion of impairments in conducting instrumental activities of daily living in case definition of dementia would have increased the sensitivity of our case-definition and would have enabled us to identify mild cases of dementia as well as the moderate to severe cases. However, this would also result in a great number of false positives. To ensure high specificity and to obtain unbiased estimates, we applied stringent criteria, requiring severe cognitive and functional impairment, for classification as dementia. As a result, only moderate to severe dementia cases are included in this study.

Assessment of Covariates in risk factor analysis

Trained interviewers asked participants about any doctor diagnosis of cardiovascular disease, stroke, diabetes, and depression as well as frequency of alcohol intake and smoking habits.[2 3] To assess participants' levels of physical activity, participants were asked how often they participated in vigorous, moderate, and mild physical activities during their leisure time. Examples for the types of activities and their associated intensities were shown to the participants to assist their response. The questions were extracted from a validated physical activity questionnaire and previously used in the Health Survey for England. A summary measure for physical activity status was compiled for each participant and categorized into sedentary, mild, moderate, and vigorous levels. [4 5]

Social class status was based on the National Statistics Socio-Economic Classification (NS-SEC) indicator and classified in 5 categories of managerial and professional; intermediate; small employers and own-account workers; lower supervisory and technical; semi-routine.[6]

Total income was calculated from all sources of income including employment, pensions, benefits, assets (interests from savings, shares, and bonds, rental income) and other sources adjusted (equivalised) for family size. Net wealth was calculated from net housing and non-housing wealth, including financial and physical wealth, equivalised for family size.[7]

Height was measured to the nearest millimetre using a free standing stadiometer and weight to the nearest 0.1 kg using portable electronic scales. Body mass index was calculated by dividing weight in kilograms over height in square meters. Systolic and diastolic blood pressure was measured using the Omron HEM-907 blood pressure monitor after the participant was seated for at least 5 minutes. Three readings were taken and the mean of the second and third reading was used for analysis.

Blood samples were taken using standard protocols and cholesterol levels were determined using the DAX oxidase assay. Detailed information on the protocols, analyses, internal and external quality control protocols are provided elsewhere.[2 8]

Section 2: Supplement Figures and Tables:

Supplement Figure 1: Flow-diagram of participants recruited to the English Longitudinal Study of Ageing (2002-2013)

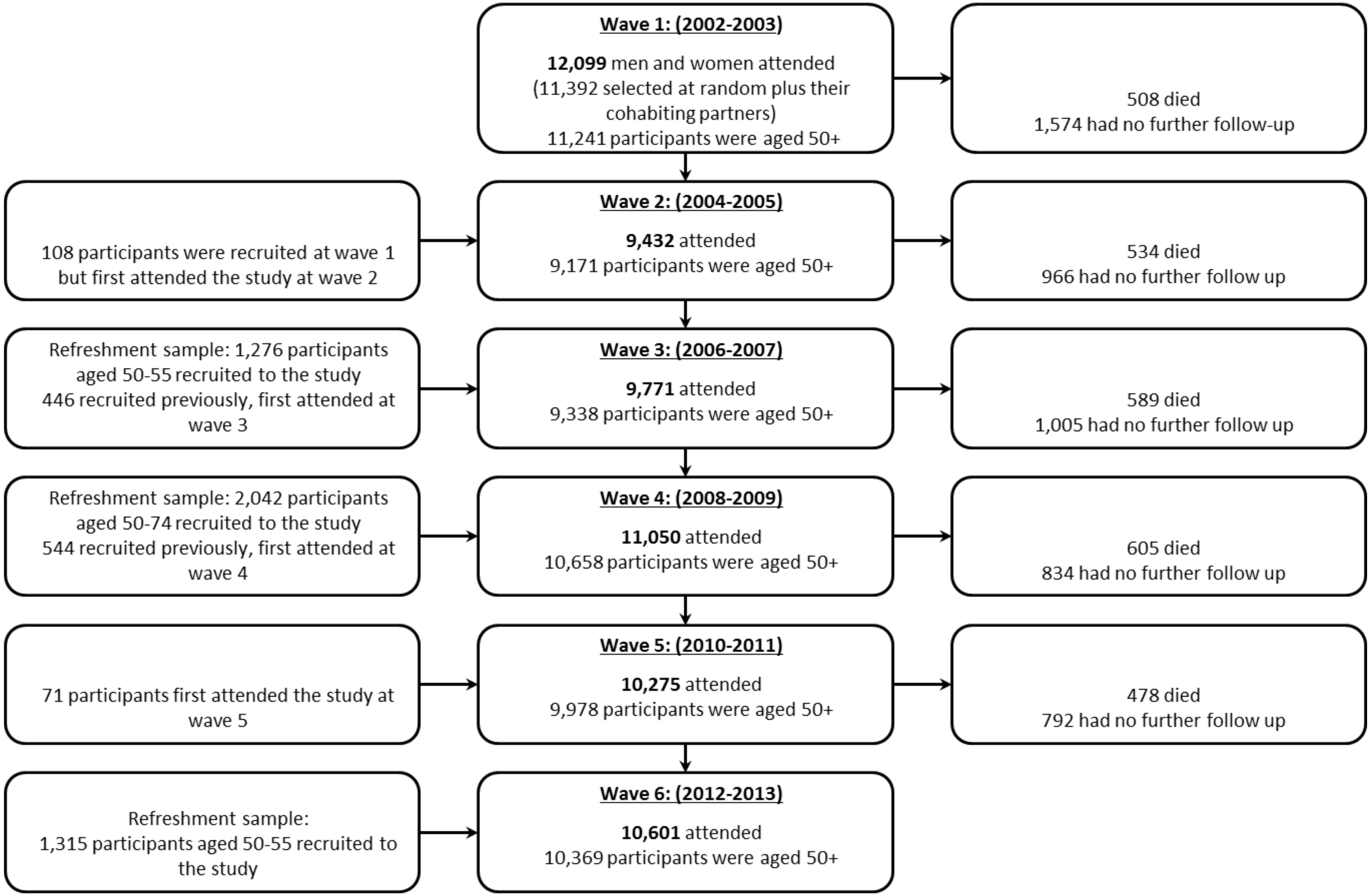

Supplement Figure 2: The prevalence of cardiovascular disease by age and sex obtained from pooling 6 waves of ELSA data (2002-2013), compared to prevalence estimates obtained at the mid-point of the time-frame (wave 3; 2006-2007).

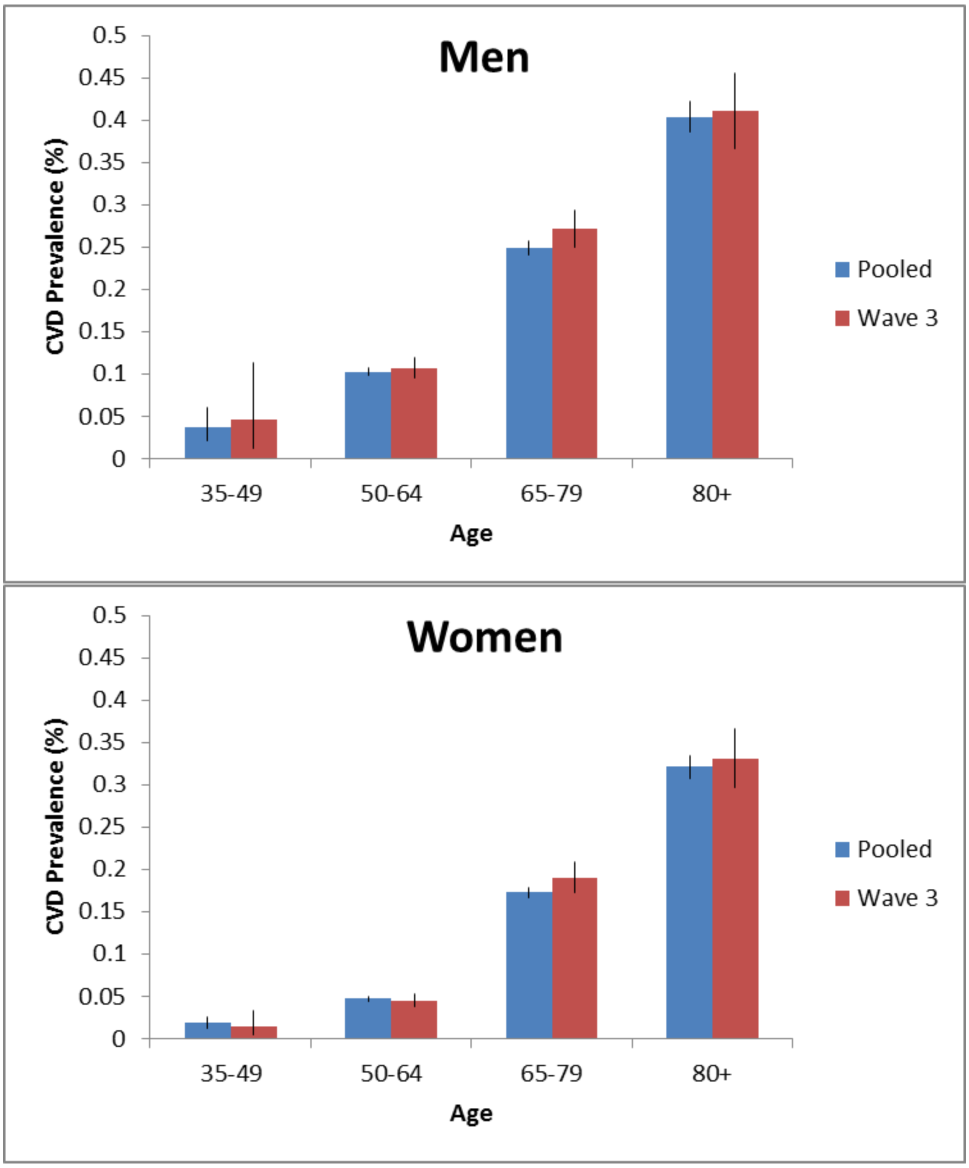

Supplement Figure 3: Observed* (1993-2013) and projected* (2014-2040) cardiovascular and non-cardiovascular mortality rates by age and sex. The mortality rates are displayed on a logarithmic scale.

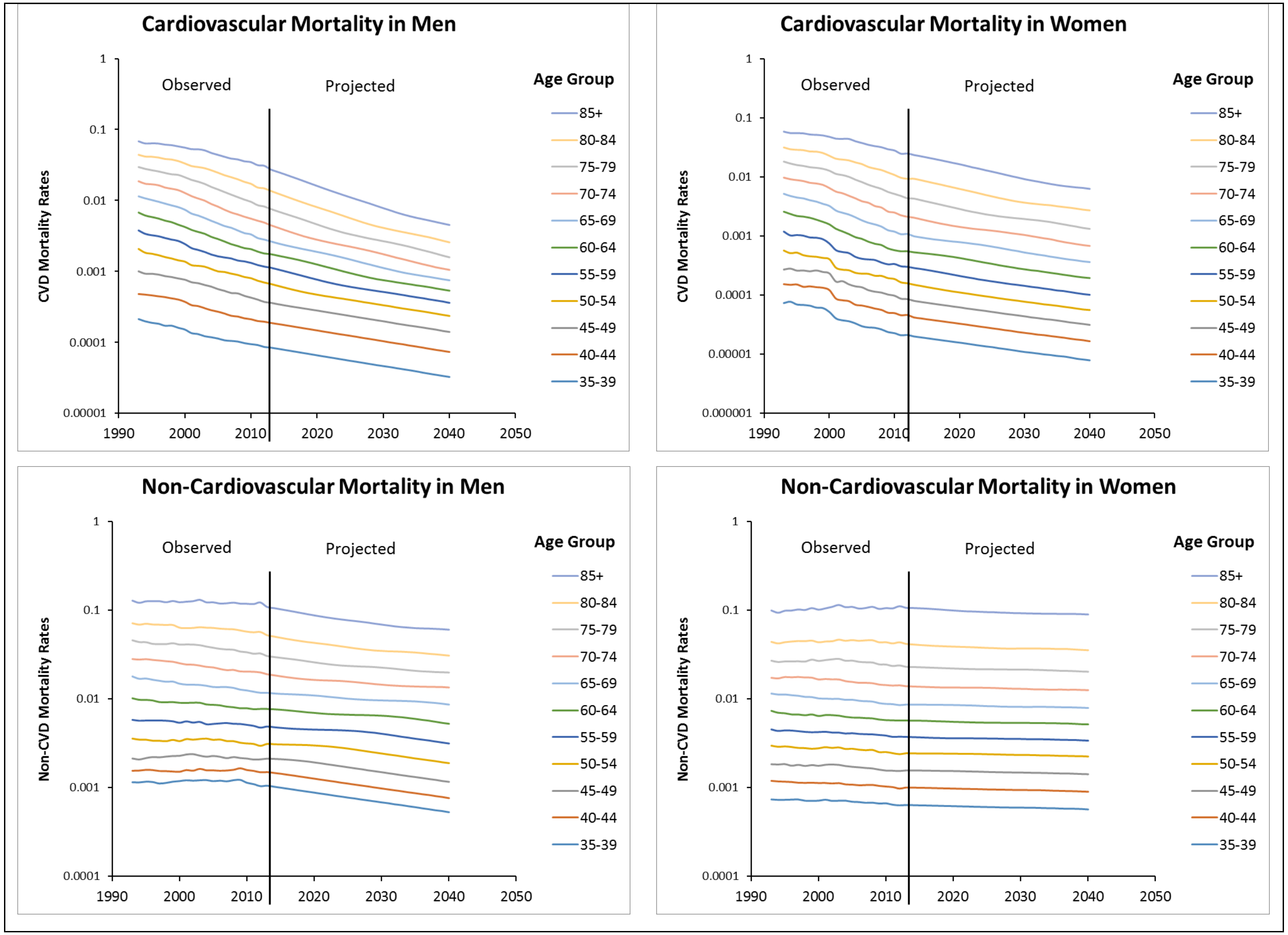

* Source of data for the observed rates is the UK Office for national statistics. Projected rates are obtained by applying Bayesian age-period-cohort models (Guzman-Castillo M, et al. Future declines of coronary heart disease mortality in England and Wales could counter the burden of population ageing. *PLoS One* 2014; 9(6):e99482).

Supplement Table 1: Odds ratio of cardiovascular and non-cardiovascular mortality for each health state in the IMPACT-BAM model obtained from the English Longitudinal Study of Ageing 2002-2013

IMPACT-BAM Health State	Cardiovascular Mortality OR (95% CI)	Non-Cardiovascular Mortality OR (95% CI)
1: Free of CVD, CI, and FI	Ref	Ref
2: CVD, no FI	2.34 (2.05, 2.62)	0.28 (0.06, 0.50)
3: CVD and CI, no FI	3.13 (2.77, 3.49)	1.07 (0.75, 1.40)
4: CI, no FI	1.60 (1.20, 2.00)	1.13 (0.91, 1.35)
5: CVD and FI	3.10 (2.79, 3.41)	1.55 (1.33, 1.77)
6: CVD, CI, and FI (Dementia)	3.71 (3.37, 4.06)	1.92 (1.65, 2.18)
7: CI and FI (Dementia)	2.20 (1.74, 2.65)	2.10 (1.88, 2.33)
8: FI without CI or FI	1.54 (1.18, 1.91)	1.32 (1.15, 1.50)

*CVD: cardiovascular disease; CI: Cognitive Impairment; FI: Functional Impairment
Coupling of cognitive and functional impairment represent dementia.

Supplement Figure 4: Age and sex standardised cardiovascular incidence and mortality rates in the English Longitudinal Study of Ageing 2002-2013

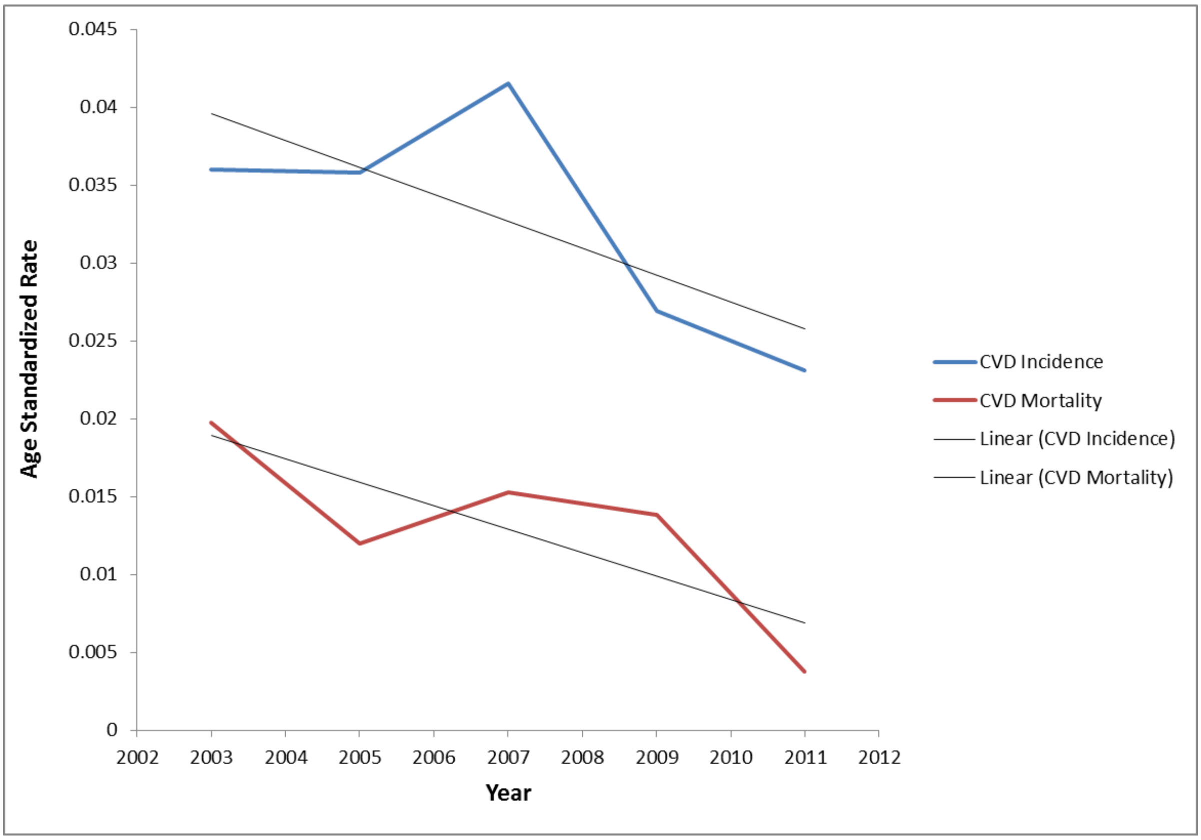

Supplement Table 2: Baseline Characteristics of the English Longitudinal Study of Ageing participants *

	Wave 1 (2002-2003) N=12,085	Wave 2 (2004-2005) N=9,424	Wave 3 (2006-2007) N=9,763	Wave 4 (2008-2009) N=10,942	Wave 5 (2010-2011) N=10,142	Wave 6 (2012-2013) N=10,594	P for temporal trend
**Age**	64.2 (11.0)	65.8 (10.6)	64.5 (11.3)	65.2 (10.3)	66.8 (10.0)	66.6 (10.3)	
**Male (%)**	5,332 (44 %)	4,124 (44 %)	4,293 (44 %)	4,862 (44 %)	4,490 (44 %)	4,742 (45 %)	
**Education**							<0.001
**No qualification**	6,034 (50.0 %)	4,414 (46.9 %)	3,630 (37.3 %)	4,154 (38.1 %)	3,918 (38.8 %)	3,910 (38.1 %)	
**A level / O level / equivalent**	3,315 (27.5 %)	2,706 (28.8 %)	3,017 (31.0 %)	3,379 (31.0 %)	3,096 (30.7 %)	3,224 (31.4 %)	
**University / Higher**	2,716 (22.5 %)	2,290 (24.3 %)	3,075 (31.6 %)	3,366 (30.1 %)	3,078 (30.5 %)	3,126 (30.5 %)	
**Social Class**							<0.001
**I/II**	2,719 (23.6 %)	1,957 (21.7 %)	1,940 (20.8 %)	2,110 (20.2 %)	1,883 (19.5 %)	1,727 (19.4 %)	
**III-M/III-NM**	5,109 (44.3 %)	3,971 (44.1 %)	4,017 (43.1 %)	4,348 (41.7 %)	4,017 (41.7 %)	3,669 (41.3 %)	
**IV/V**	3,719 (32.2 %)	3,082 (34.2 %)	3,356 (36.0 %)	3,967 (38.1 %)	3,736 (38.8 %)	3,496 (39.3 %)	
**Current Smoker (%)**	2,159 (18.2 %)	1,472 (15.6 %)	1,507 (15.4 %)	1,548 (14.2 %)	1,315 (13.0 %)	1,199 (11.3 %)	<0.001
**Daily alcohol intake (%)**	3,318 (27.9 %)	3,185 (33.8 %)	3,204 (32.8 %)	3,726 (35.9 %)	3,466 (34.2 %)	3,516 (33.2 %)	<0.001
**Sedentary or low Physical activity (%)**	2,789 (33.2 %)	2,573 (27.3 %)	2,947 (30.2 %)	3,374 (30.9 %)	3,219 (31.7 %)	2,756 (26.0 %)	<0.001
**Body Mass Index (Kg/m2)**	-	27.9 (4.9)	-	28.3 (5.3)	-	28.2 (5.1)	<0.001
**Systolic Blood Pressure mmHg**	-	135.3 (18.9)	-	132.7 (17.8)	-	132.0 (17.5)	<0.001
**Diastolic Blood Pressure mmHg**	-	75.0 (11.2)	-	74.3 (11.0)	-	73.6 (10.8)	<0.001
**LDL Cholesterol (mmol/l)**	-	3.59 (1.00)	-	3.27 (1.03)	-	3.22 (1.04)	<0.001
**HDL Cholesterol (mmol/l)**	-	1.52 (0.39)	-	1.56 (0.4)	-	1.66 (0.48)	<0.001
**Diabetes (%)**	866 (7.2 %)	809 (8.6 %)	917 (9.4 %)	1,086 (9.9 %)	1,186 (11.7 %)	1,285 (12.1 %)	<0.001
**Cardiovascular Disease (%)**	1,804 (14.9 %)	1,565 (16.6 %)	1,489 (15.3 %)	1,526 (14.0 %)	1,579 (15.6 %)	1,556 (14.7 %)	<0.001
**Cerebrovascular Disease/Stroke (%)**	516 (4.3 %)	475 (5.0 %)	474 (4.9 %)	506 (4.6 %)	517 (5.1 %)	537 (5.1 %)	0.34

* Values are mean (standard deviation) or number (%)

Supplement Table 3: Number of Incident cases of dementia at each wave of the English Longitudinal Study of Ageing (2002 – 2013)

	Age	Wave 2 (2004 - 2005)	Wave 3 (2006 - 2007)	Wave 4 (2008 - 2009)	Wave 5 (2010 - 2011)	Wave 6 (2012 - 2013)
**All**		*N = 9,225*	*N = 8,155*	*N = 8,386*	*N = 9,767*	*N = 8,941*
	**50 - 54**	13 (0.7 %)	4 (0.4 %)	2 (0.1 %)	4 (0.3 %)	1 (0.2 %)
	**55 - 59**	14 (0.8 %)	6 (0.3 %)	8 (0.5 %)	6 (0.3 %)	6 (0.3 %)
	**60 - 64**	14 (1.0 %)	8 (0.6 %)	8 (0.6 %)	8 (0.4 %)	5 (0.3 %)
	**65 - 69**	27 (1.9 %)	11 (0.8 %)	15 (1.3 %)	15 (1.0 %)	14 (0.9 %)
	**70 - 74**	44 (3.8 %)	19 (1.8 %)	19 (1.8 %)	22 (1.6 %)	14 (1.1 %)
	**75 - 79**	32 (4.0 %)	28 (3.5 %)	38 (4.8 %)	35 (4.3 %)	23 (2.6 %)
	**80 - 84**	47 (8.6 %)	47 (8.9 %)	34 (7.1 %)	35 (7.4 %)	12 (2.4 %)
	**85- 89**	18 (10.6 %)	27 (13.4 %)	33 (15.1 %)	26 (11.0 %)	20 (8.3 %)
	**90 +**	11 (25.0 %)	10 (20.0 %)	12 (27.3 %)	16 (30.8 %)	3 (4.8 %)
	Total	**220 (2.4 %)**	**160 (2.0 %)**	**169 (2.0 %)**	**167 (1.7 %)**	**98 (1.1 %)**
**Men**		*N = 4,145*	*N = 3,611*	*N = 3,754*	*N = 4,402*	*N = 3,986*
	**50 - 54**	5 (0.6 %)	2 (0.5 %)	2 (0.3 %)	1 (0.2 %)	0
	**55 - 59**	4 (0.5 %)	4 (0.5 %)	4 (0.5 %)	2 (0.2 %)	2 (0.2 %)
	**60 - 64**	9 (1.3 %)	6 (0.9 %)	3 (0.4 %)	5 (0.5 %)	2 (0.2 %)
	**65 - 69**	12 (1.9 %)	7 (1.1 %)	5 (0.9 %)	8 (1.2 %)	7 (1.0 %)
	**70 - 74**	21 (4.0 %)	13 (2.6 %)	10 (2.0 %)	10 (1.6 %)	12 (2.0 %)
	**75 - 79**	15 (4.2 %)	11 (3.2 %)	18 (5.4 %)	19 (4.9 %)	9 (2.3 %)
	**80 - 84**	22 (11.4 %)	14 (6.9 %)	13 (6.4 %)	13 (6.3 %)	7 (3.4 %)
	**85- 89**	5 (7.1 %)	15 (19.5 %)	10 (14.5 %)	5 (6.1 %)	7 (6.5 %)
	**90 +**	3 (17.7 %)	2 (14.3 %)	2 (13.3 %)	8 (42.1 %)	1 (5.3 %)
	Total	**96 (2.3 %)**	**74 (2.1 %)**	**67 (1.8 %)**	**71 (1.6 %)**	**47 (1.2 %)**
**Women**		*N = 5,080*	*N = 4,544*	*N = 4,632*	*N = 5,365*	*N = 4,955*
	**50 - 54**	8 (0.8 %)	2 (0.3 %)	0	3 (0.4 %)	1 (0.3 %)
	**55 - 59**	10 (1.0 %)	2 (0.2 %)	4 (0.4 %)	4 (0.4 %)	4 (0.4 %)
	**60 - 64**	5 (0.7 %)	2 (0.3 %)	5 (0.7 %)	3 (0.3 %)	3 (0.3 %)
	**65 - 69**	15 (2.0 %)	4 (0.6 %)	10 (1.6 %)	7 (0.9 %)	7 (0.9 %)
	**70 - 74**	23 (3.6 %)	6 (1.1 %)	9 (1.6 %)	12 (1.6 %)	2 (0.3 %)
	**75 - 79**	17 (3.8 %)	17 (3.7 %)	20 (4.4 %)	16 (3.7 %)	14 (2.8 %)
	**80 - 84**	25 (7.1 %)	33 (10.1 %)	21 (7.6 %)	22 (8.2 %)	5 (1.7 %)
	**85- 89**	13 (13.0 %)	12 (9.7 %)	23 (15.4 %)	21 (13.6 %)	13 (9.6 %)
	**90 +**	8 (29.6 %)	8 (22.2 %)	10 (34.5 %)	8 (24.2 %)	2 (4.7 %)
	Total	**124 (2.4 %)**	**86 (1.9 %)**	**102 (2.2 %)**	**96 (1.8 %)**	**51 (1.0 %)**

Supplement Figure 5: Comparison of incidence (per 1000 person years) of dementia in English Longitudinal Study of Ageing (waves 4 to 6 (2008 – 2013)) with Cognitive Function and Ageing Study II (2008 – 2011) *

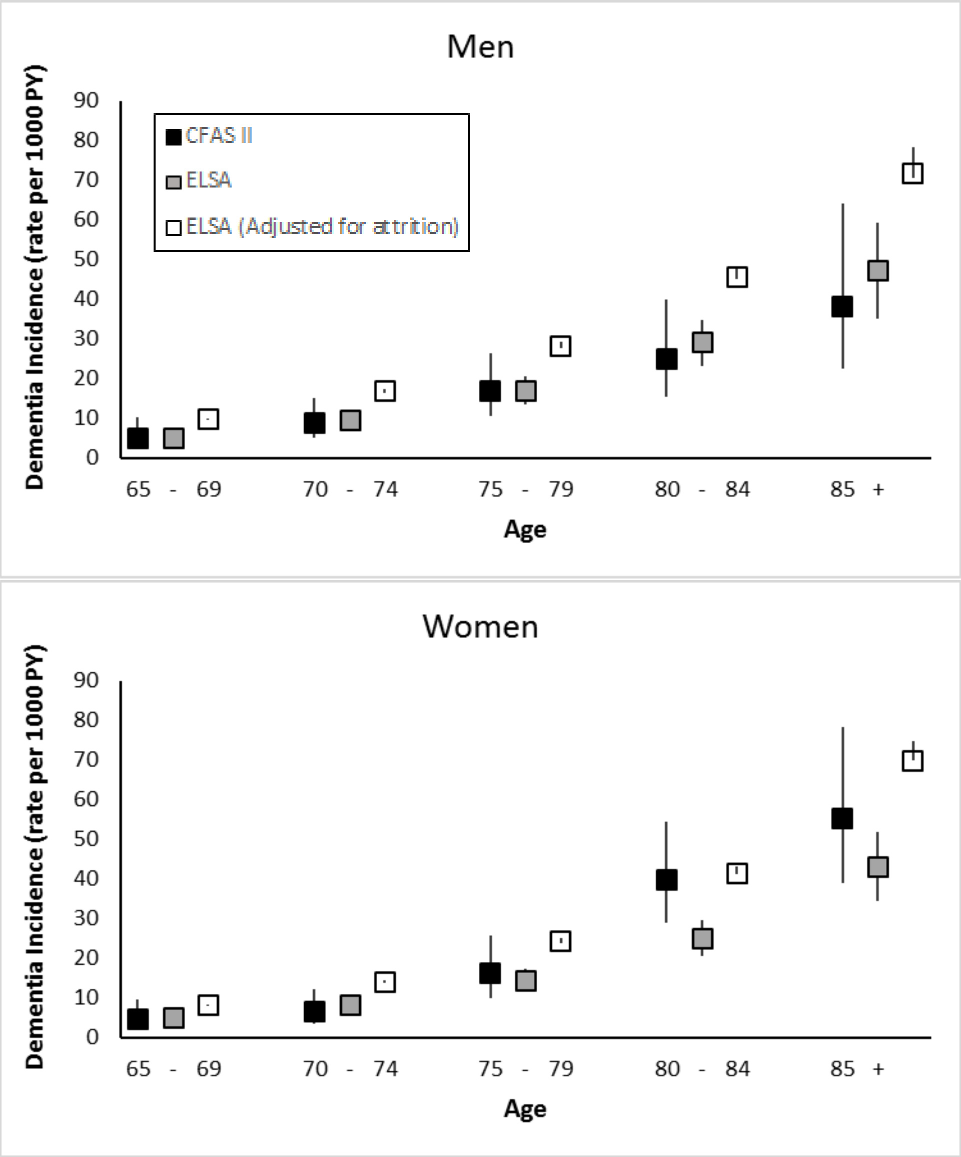

* Data for dementia incidence in the UK was available for persons aged 65+ from CFAS-II study between years 2008-2011. For comparability, we display estimates obtained from ELSA corresponding to the similar time-frame and age group.

Supplement Figure 6: Age-sex specific trends in incidence of dementia for years 2005, 2010, 2015 corrected for dropouts using joint models in the English Longitudinal Study of Ageing.

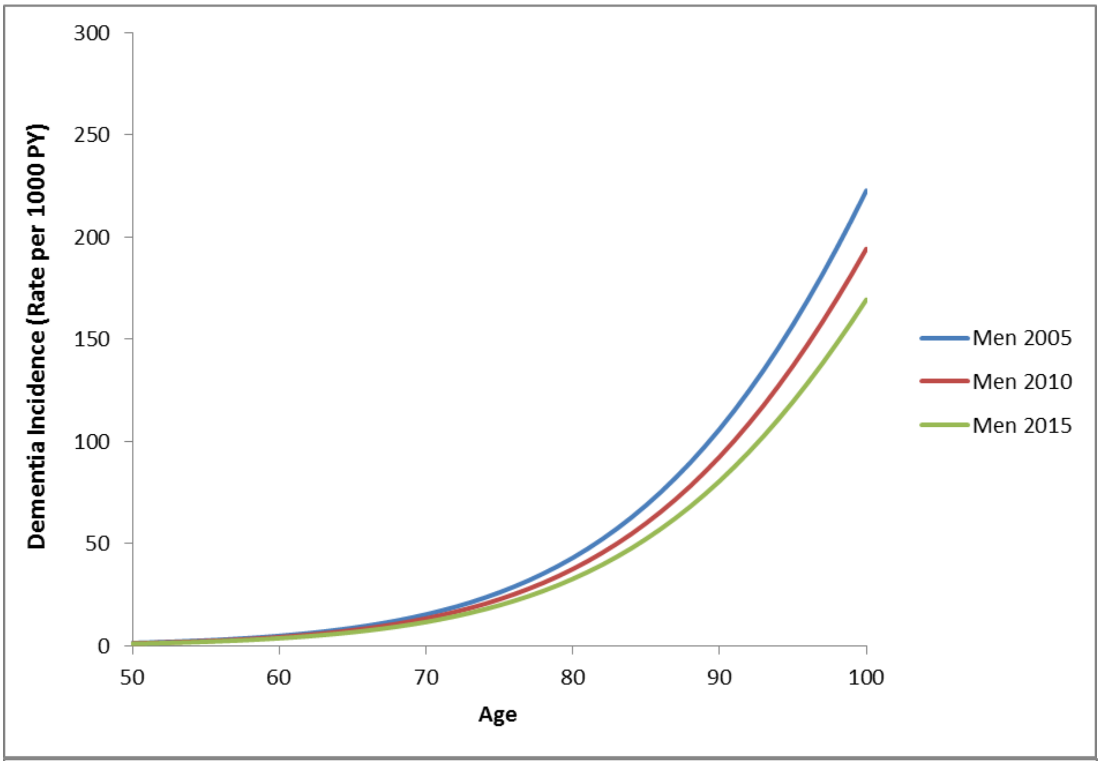

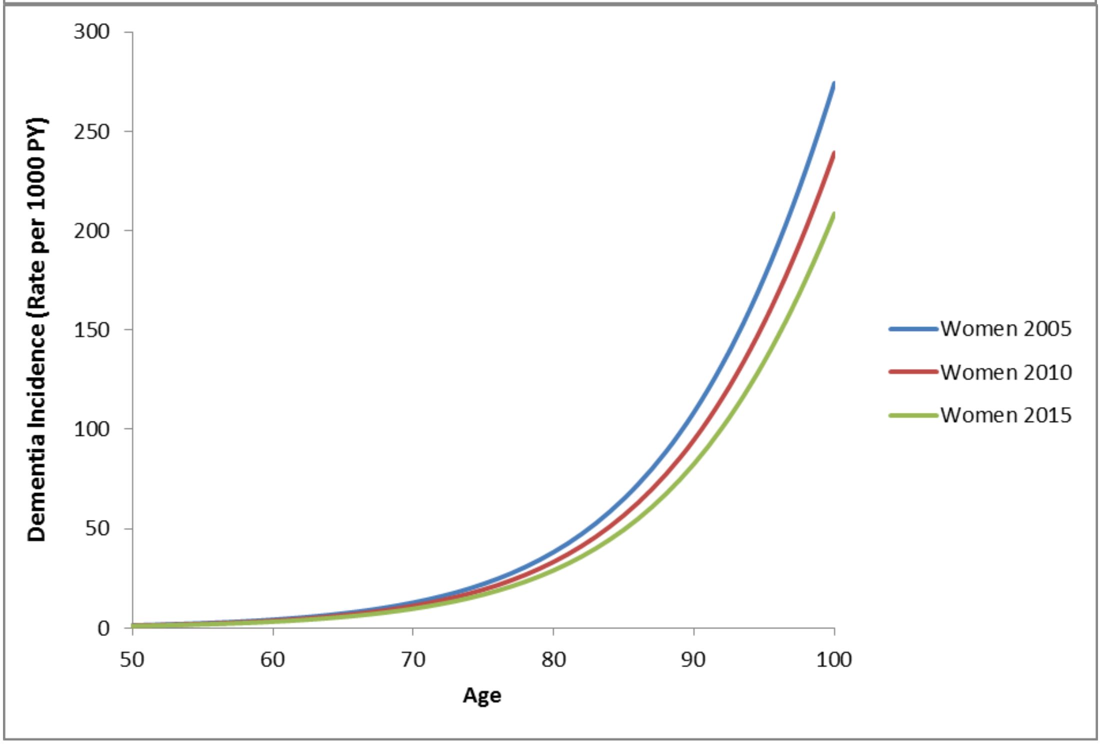

Supplement Figure 7: Incidence of dementia as observed and corrected for dropouts using joint models, at mid-point of ELSA data collection period (2002-2013).

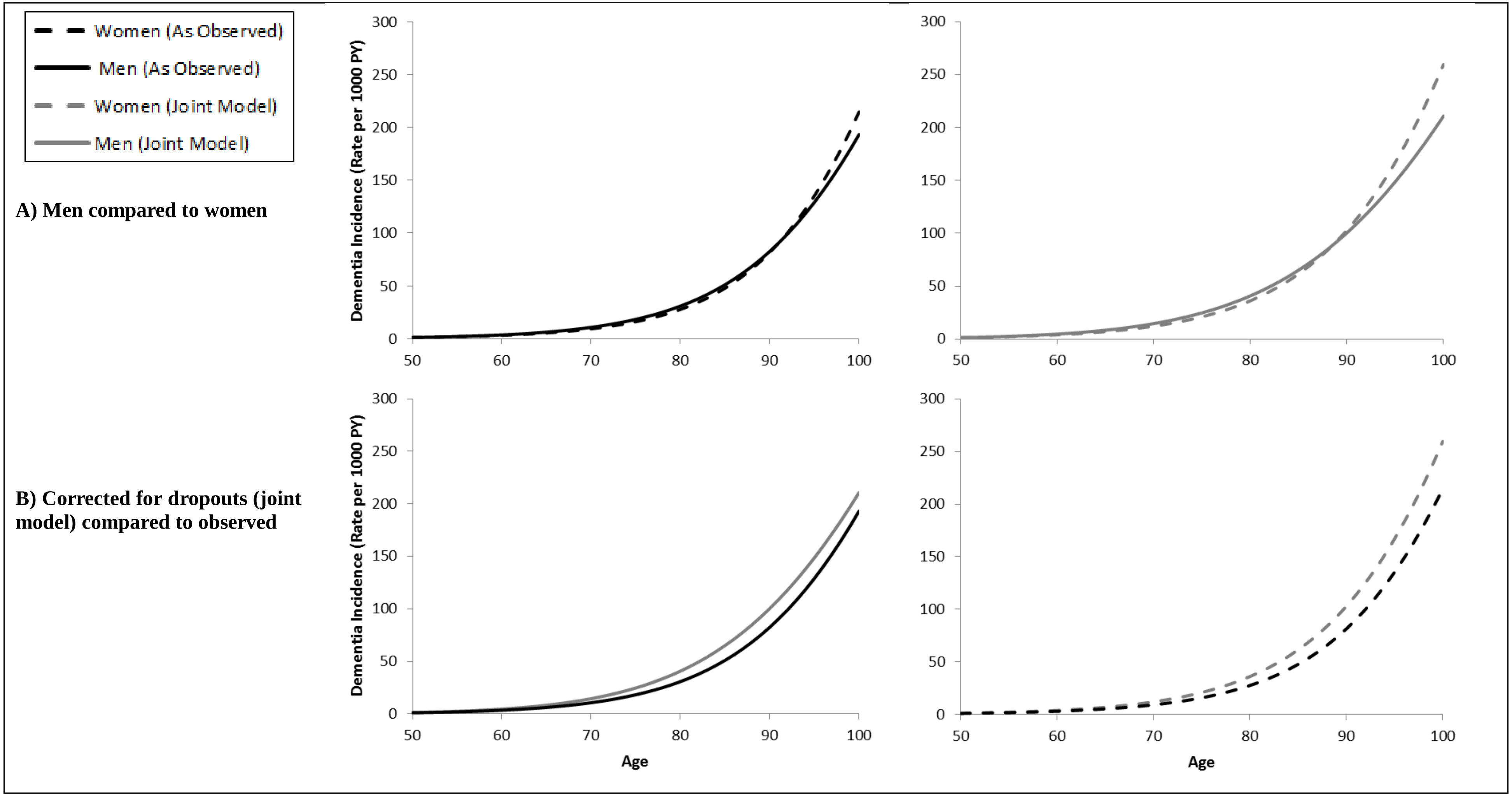

Supplement Figure 8: Incidence of dementia A) as observed and B) corrected for dropouts using joint models, for years 2005 and 2010 in men and women

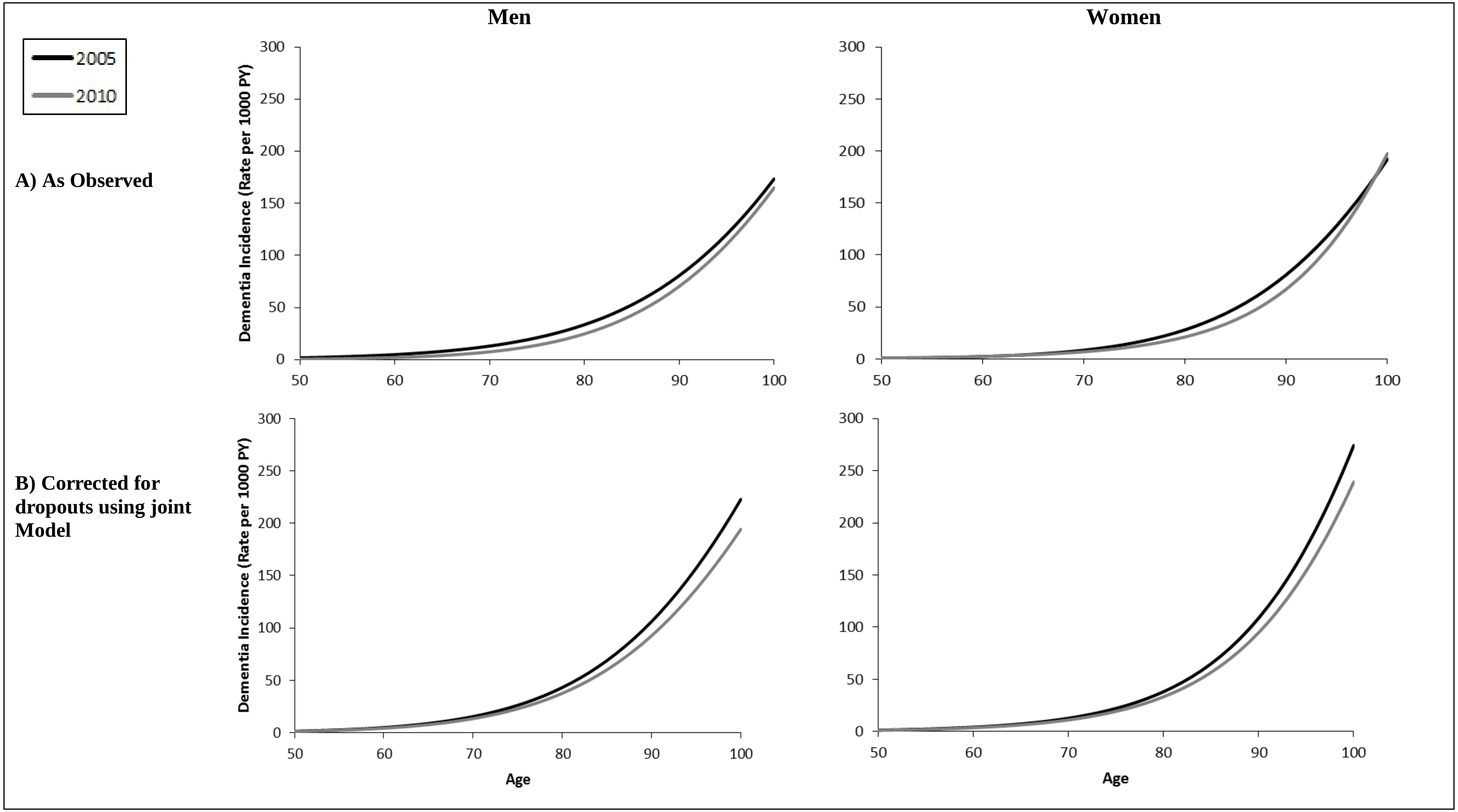

Supplement Table 4: Calendar trend in dementia incidence adjusted for change in level of risk factors.

	Annual Change in Incident Dementia (2002 - 2013)	
	**Odds Ratio (95% CI)**	**Relative Annual Change (%) (95% CI)**
**Calendar Trend (per year)**	0.973 (0.971, 0.976)	-2.7 (-2.9, -2.4) %
**Adjusted for**		
Education	0.977 (0.974, 0.979)	-2.3 (-2.6, -2.1) %
Physical Activity	0.983 (0.980, 0.985)	-1.7 (-2.0, -1.5) %
Smoking	0.967 (0.964, 0.970)	-3.3 (-3.6, -3.0) %
Alcohol Intake	0.976 (0.973, 0.979)	-2.4 (-2.7, -2.1) %
Body Mass Index	0.978 (0.975, 0.980)	-2.2 (-2.5, -2.0) %
Blood Pressure	0.978 (0.975, 0.981)	-2.2 (-2.5, -1.9) %
LDL and HDL Cholesterol	0.979 (0.977, 0.982)	-2.1 (-2.3, -1.8) %
Diabetes	0.972 (0.970, 0.975)	-2.8 (-3.0, -2.5) %
Stroke	0.973 (0.970 - 0.976)	-2.7 (-3.0, -2.4) %
Depression	0.974 (0.971 - 0.977)	-2.7 (-2.9, -2.3) %
Social Class	0.971 (0.968 - 0.974)	-3.0 (-3.2, -2.6) %
Total Income and Net Wealth	0.976 (0.974 - 0.979)	-2.4 (-2.6, -2.1) %
Multivariable Adjusted	0.980 (0.977 - 0.982)	-2.0 (-2.3, -1.7) %

Supplement Table 5: Observed prevalence (%) of dementia (95% confidence interval) by age-group at each wave of data collection for English Longitudinal Study of Ageing (ELSA) 2002-2013.

	Age	Prevalence % (95 % Confidence Interval)					
		**Wave 1 (2002-2003)**	**Wave 2 (2004-2005)**	**Wave 3 (2006-2007)**	**Wave 4 (2008-2009)**	**Wave 5 (2010-2011)**	**Wave 6 (2012-2013)**
**Men**							
	50 - 54	2.0 (1.1, 2.9)	1.9 (0.8, 3.7)	1.0 (0.3, 1.8)	0.8 (0.2, 1.9)	1.3 (0.0, 3.0)	1.1 (0.0, 2.3)
	55 - 59	1.7 (0.9, 2.4)	1.6 (0.9, 2.7)	1.2 (0.5, 1.9)	1.4 (0.7, 2.4)	1.1 (0.4, 1.8)	1.3 (0.5, 2.0)
	60 - 64	3.1 (1.9, 4.3)	1.2 (0.5, 2.3)	1.4 (0.5, 2.3)	1.4 (0.8, 2.3)	2.0 (1.1, 2.9)	1.1 (0.4, 1.8)
	65 - 69	4.1 (2.7, 5.5)	3.6 (2.3, 5.3)	2.3 (1.1, 3.5)	2.5 (1.5, 3.9)	2.1 (1.1, 3.1)	1.9 (1.0, 2.7)
	70 - 74	4.0 (2.5, 5.4)	4.4 (2.9, 6.4)	4.8 (3.0, 6.7)	3.5 (2.2, 5.1)	2.5 (1.3, 3.6)	3.3 (1.9, 4.7)
	75 - 79	8.0 (5.6, 10.4)	6.7 (4.5, 9.4)	4.3 (2.3, 6.4)	6.2 (4.2, 8.8)	6.9 (4.6, 9.1)	5.1 (3.3, 7.0)
	80 - 84	10.4 (7.0, 13.8)	9.9 (6.6, 14.0)	11.2 (7.5, 15.0)	9.5 (6.3, 13.6)	12.1 (8.3, 16.0)	6.5 (3.8, 9.3)
	85 - 90	15.1 (9.1, 21.1)	20.7 (13.8, 29.0)	19.5 (12.7, 26.4)	16.8 (10.8, 24.3)	14.9 (9.2, 20.6)	13.5 (8.2, 18.8)
	90 +	15.8 (3.6, 27.9)	20.6 (8.7, 37.9)	27.3 (13.6, 41.0)	31.1 (18.2, 46.6)	31.5 (18.7, 44.3)	23.0 (12.1, 33.8)
**Women**							
	50 - 54	0.9 (0.3, 1.4)	1.0 (0.4, 2.1)	1.1 (0.4, 1.7)	0.6 (0.2, 1.5)	1.1 (0.0, 2.1)	0.9 (0.1, 1.6)
	55 - 59	1.8 (1.0, 2.6)	1.3 (0.7, 2.2)	1.2 (0.5, 1.8)	0.6 (0.2, 1.3)	0.6 (0.2, 1.1)	1.2 (0.5, 1.9)
	60 - 64	1.9 (1.0, 2.8)	1.7 (0.9, 2.9)	0.5 (0.0, 1.0)	0.8 (0.4, 1.5)	0.6 (0.2, 1.1)	0.7 (0.2, 1.1)
	65 - 69	3.6 (2.4, 4.8)	1.7 (0.9, 3.0)	1.2 (0.4, 2.1)	2.5 (1.5, 3.8)	1.8 (0.9, 2.7)	1.4 (0.6, 2.1)
	70 - 74	5.1 (3.6, 6.7)	3.7 (2.4, 5.4)	2.8 (1.5, 4.1)	2.3 (1.3, 3.6)	2.4 (1.3, 3.4)	1.6 (0.6, 2.5)
	75 - 79	8.1 (5.9, 10.2)	5.3 (3.6, 7.5)	4.6 (2.8, 6.3)	5.1 (3.3, 7.4)	4.5 (2.8, 6.2)	4.4 (2.8, 5.9)
	80 - 84	10.8 (8.1, 13.6)	10.3 (7.6, 13.6)	11.5 (8.1, 14.8)	12.4 (9.2, 16.3)	12.0 (8.7, 15.2)	7.5 (4.9, 10.2)
	85 - 90	23.2 (17.3, 29.0)	15.7 (11.0, 21.4)	23.5 (18.1, 28.8)	20.7 (15.9, 26.3)	23.1 (17.6, 28.5)	14.4 (9.6, 19.1)
	90 +	34.2 (23.3, 45.1)	28.1 (17.6, 40.8)	34.4 (24.4, 44.5)	37.1 (27.1, 48.0)	41.4 (32.3, 50.5)	28.3 (20.9, 35.7)
**Age, sex standardised**		4.5 (3.0, 6.0)	3.7 (2.3, 5.6)	3.5 (2.0, 4.9)	3.4 (2.2, 5.1)	3.5 (2.1, 4.9)	2.7 (1.5, 3.9)

Supplement Figure 9: Age- and sex-standardised prevalence of dementia at each data collection wave of the English Longitudinal Study of Ageing (ELSA) 2002-2013.

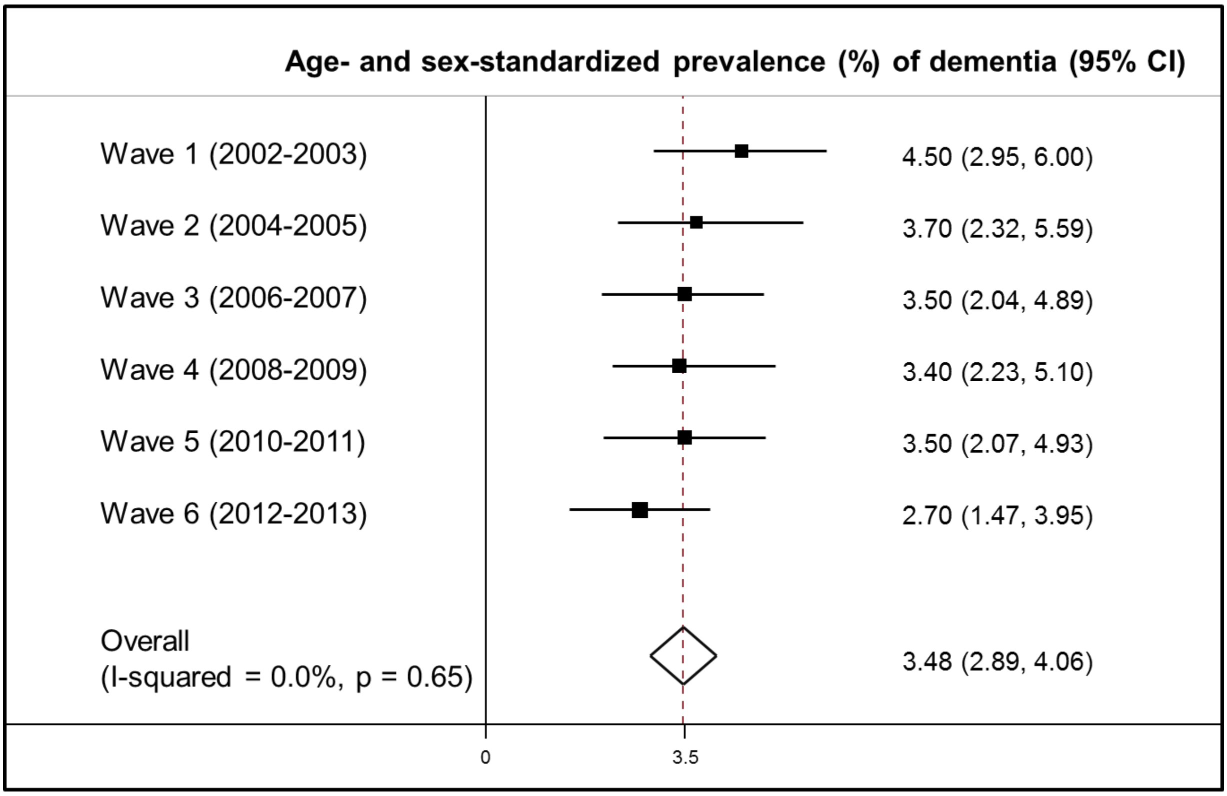

Supplement Figure 10: IMPACT-BAM predicted estimates for prevalence of dementia compared with estimates from the Cognitive Function and Ageing Study (CFAS II) in 2011

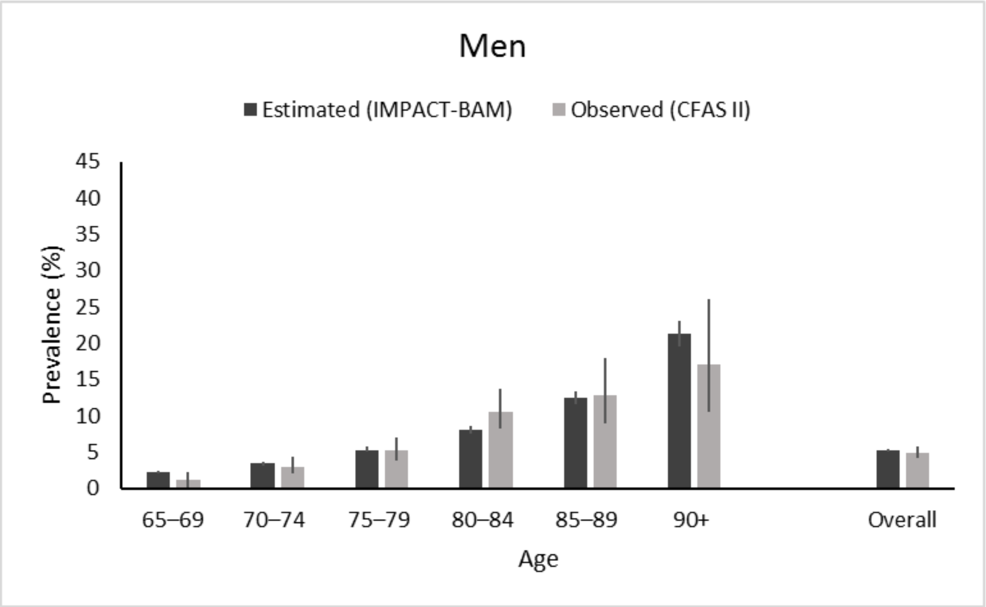

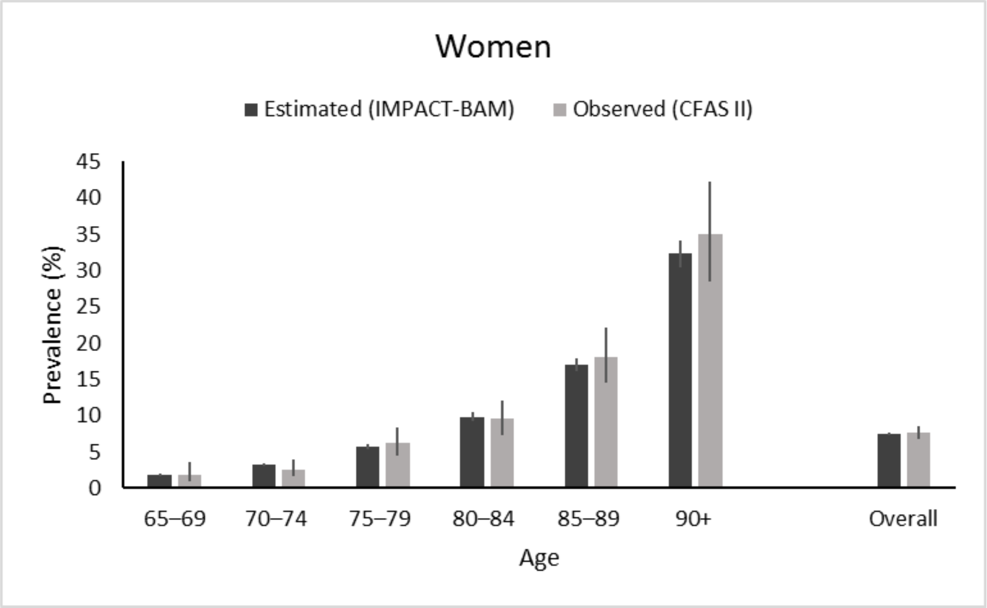

Error bars represent 95 % uncertainty intervals for estimates from IMPACT-BAM, and 95% confidence intervals for estimates from CFAS II.

Supplement Figure 11: IMPACT-BAM predicted prevalence of dementia in men and women compared to estimates from wave 7 of the English Longitudinal Study of Ageing (2014-2015)*

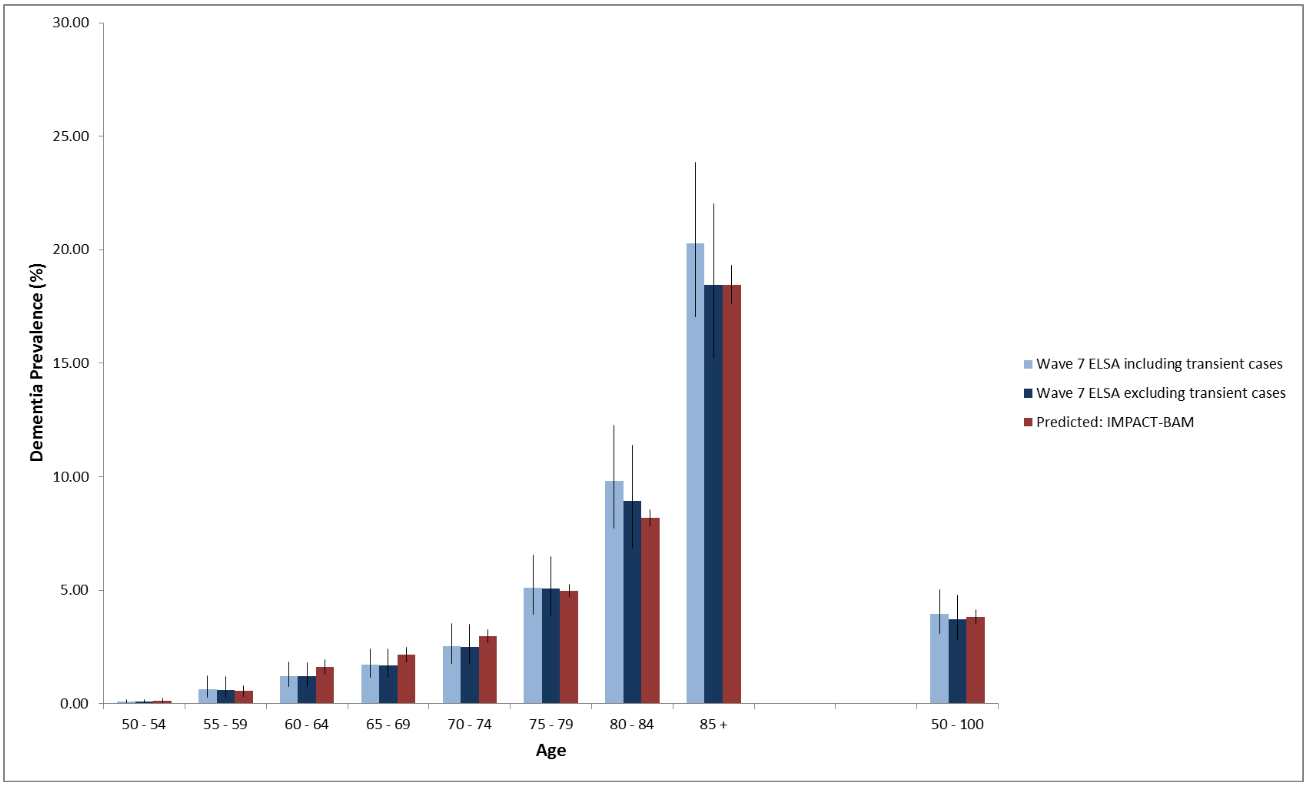

*In assessment of cognitive and functional impairment at wave 7, we were unable to exclude cases of transient cognitive or functional impairment due to absence of data at later (post wave 7) dates. We calculated the numbers of persons who would have been marked as dementia based on transient impairment in cognition and function at previous waves of ELSA and applied the age-specific proportion to the dementia prevalence at wave 7 to correct for the false positives.

Supplement Figure 12: IMPACT-BAM predicted cardiovascular disease prevalence compared with observed estimates from the Health Survey for England in 2011.

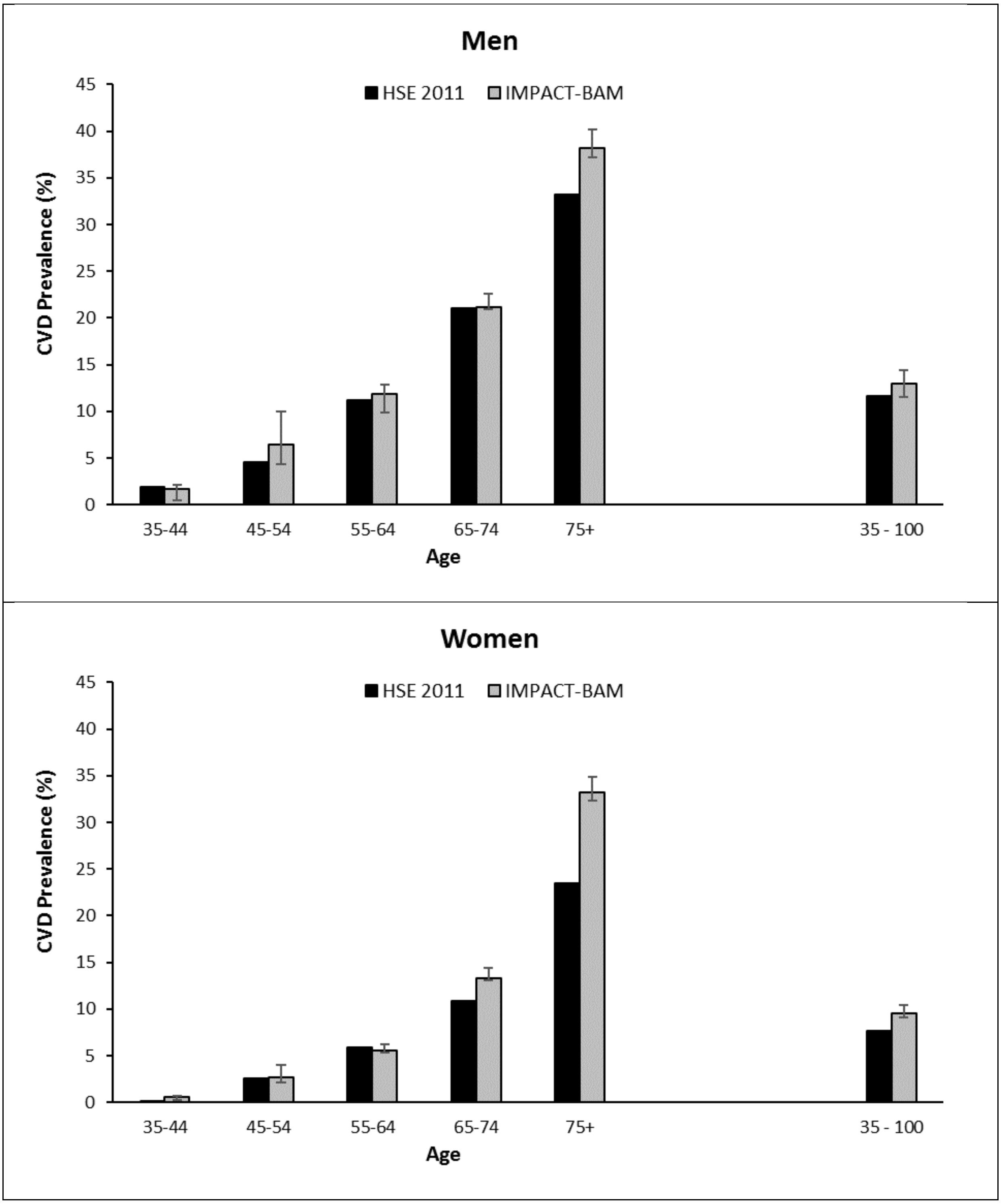

Error bars represent 95% uncertainty intervals.

Supplement Figure 13: IMPACT-BAM predicted mortality compared with observed estimates from the UK Office for National Statistics.

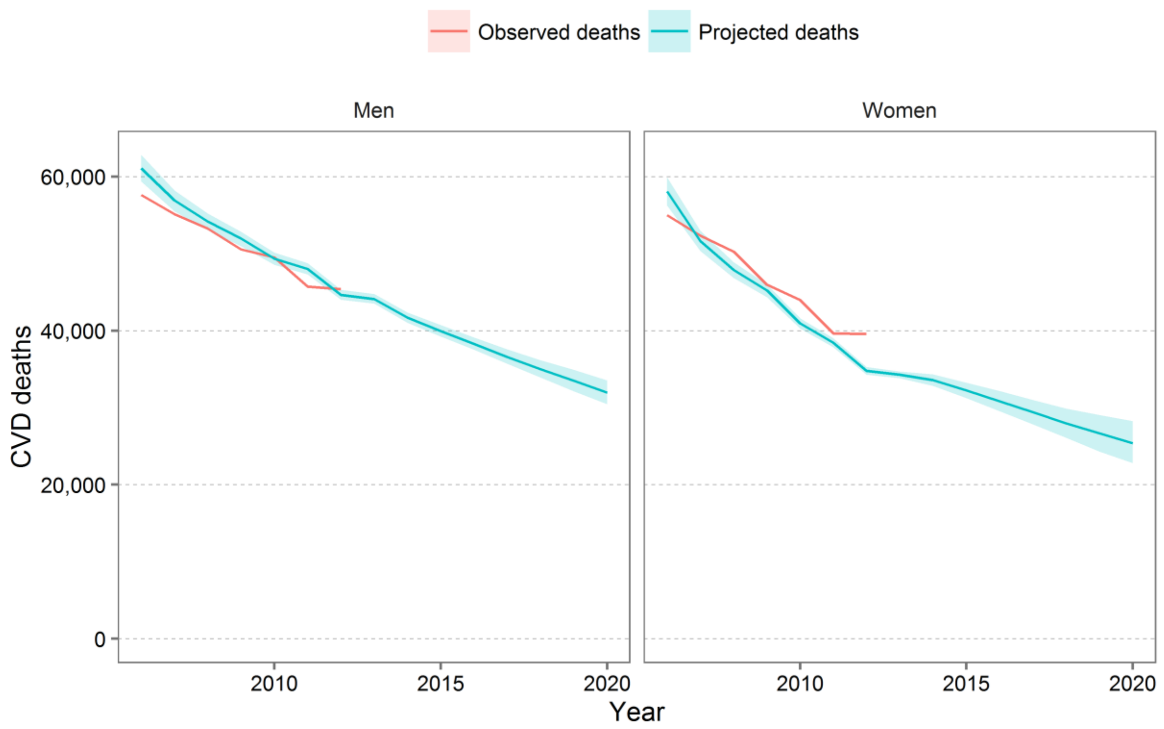

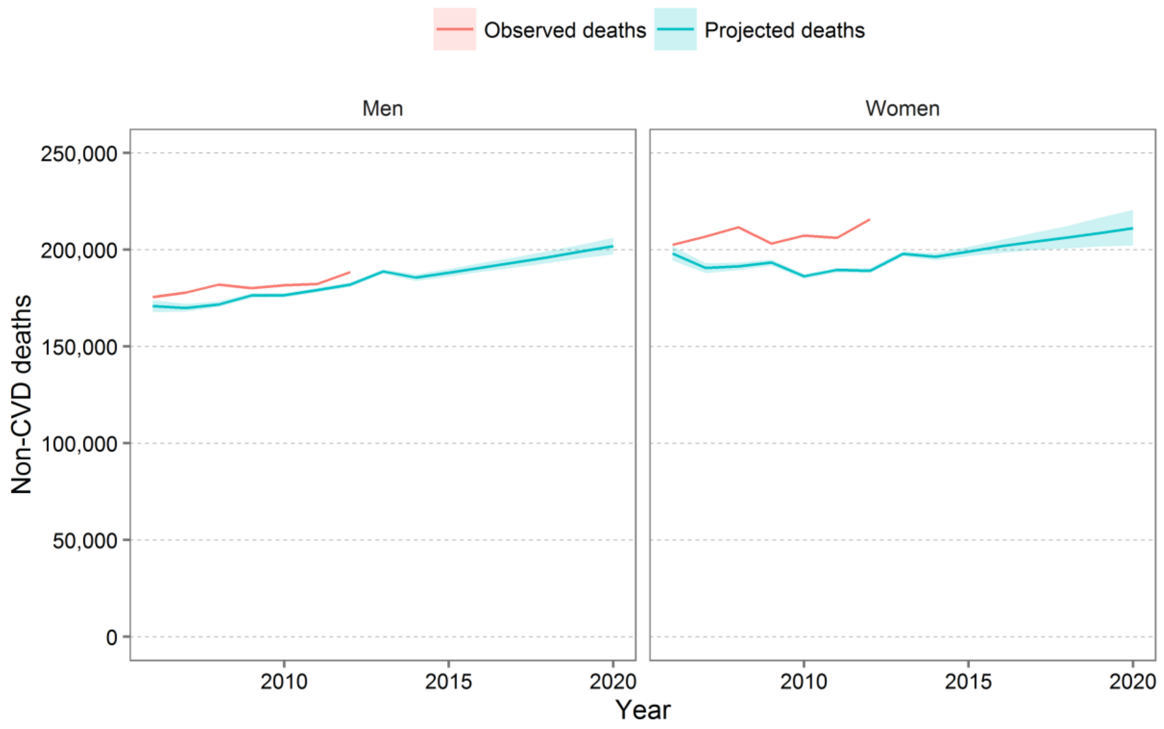

Supplement Figure 14: Age and sex specific estimated number of cases of dementia in men and women 2010-2040.

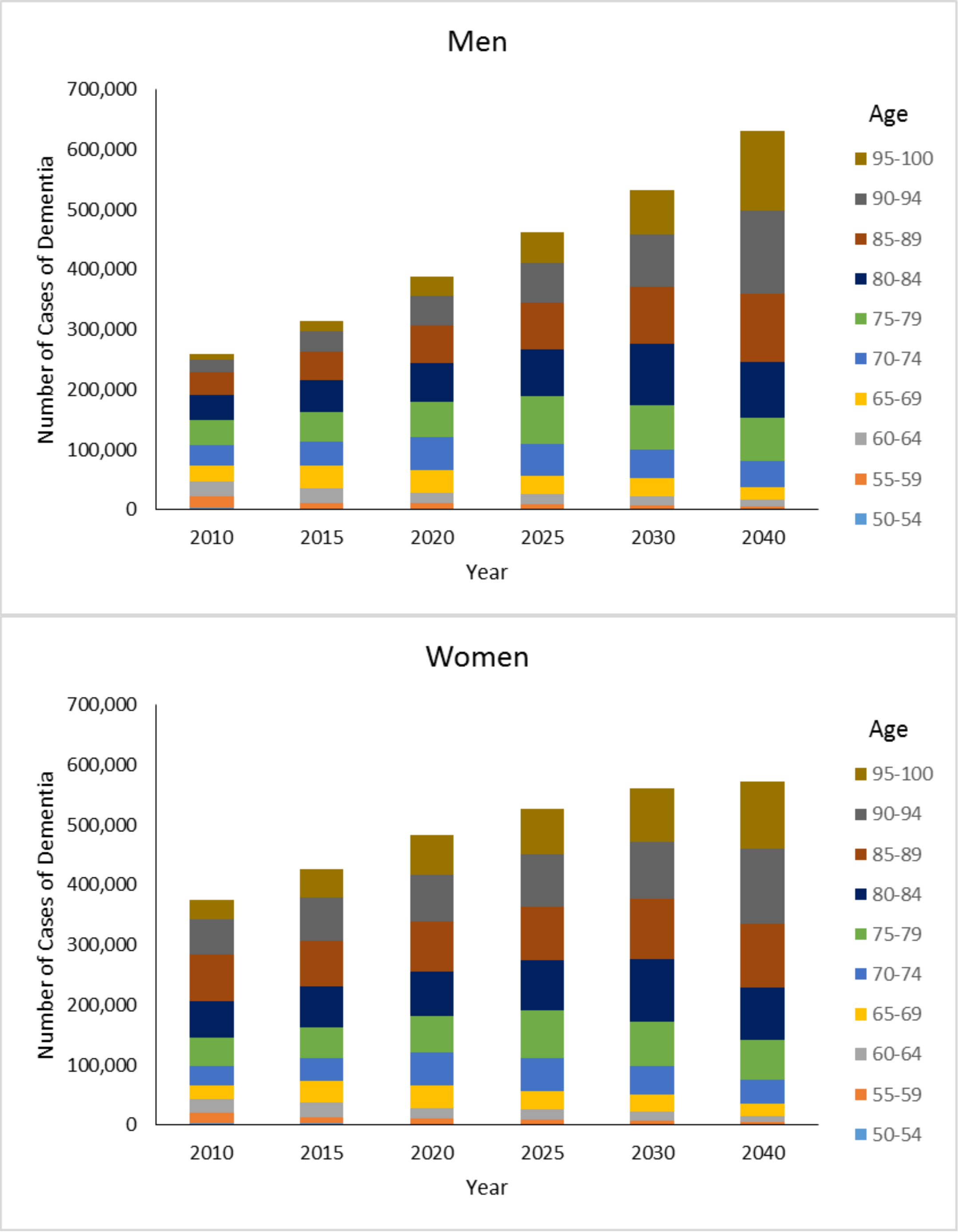

Supplement Figure 15: Sensitivity analysis for prevalence of dementia assuming no calendar trend in incidence of dementia, standardized to the population of England and Wales in 2015.

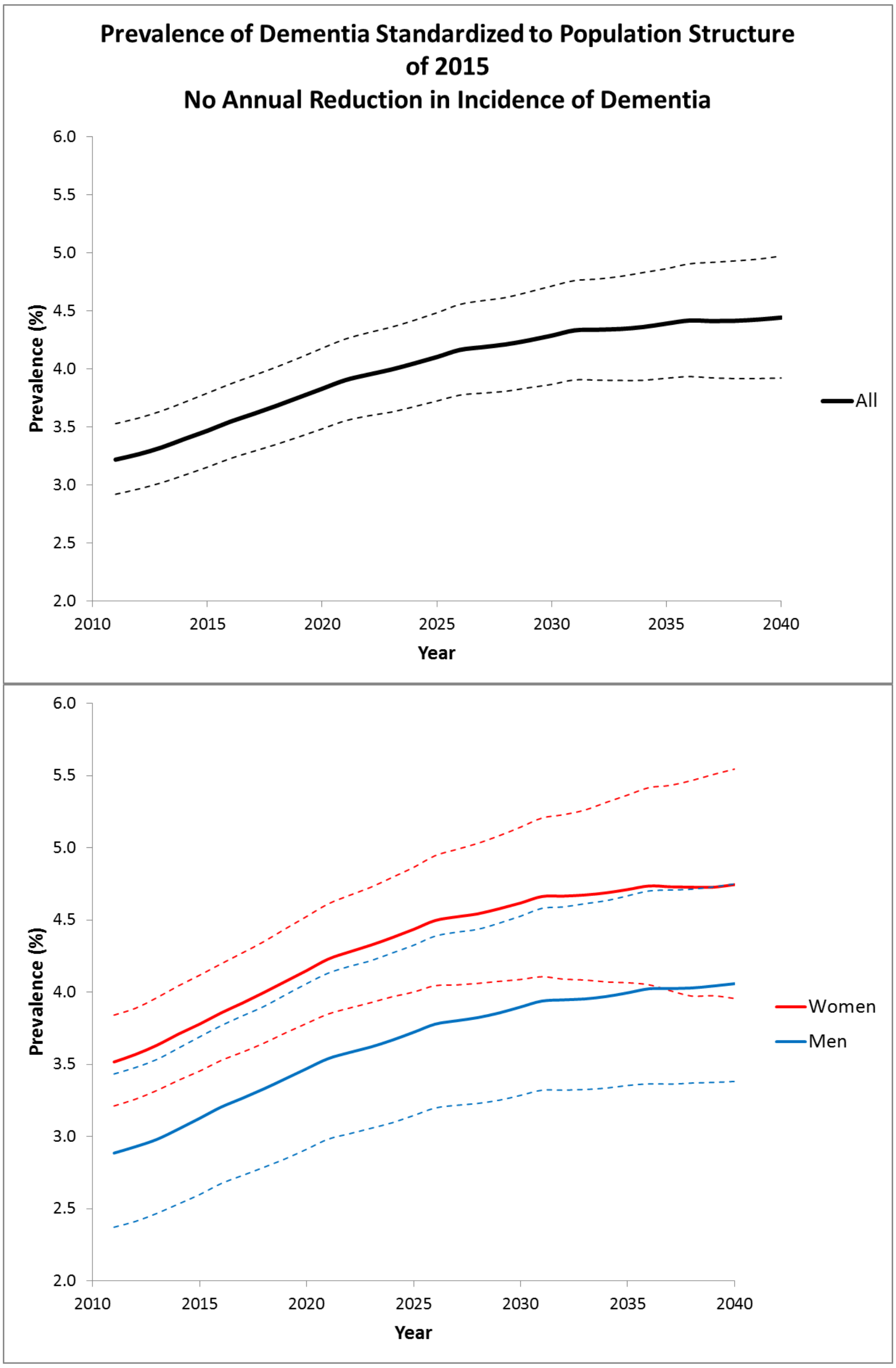

Dashed lines represent 95% uncertainty intervals.

Supplement Figure 16: Sensitivity analysis for numbers of cases of dementia assuming cardiovascular incidence does not decline after 2014

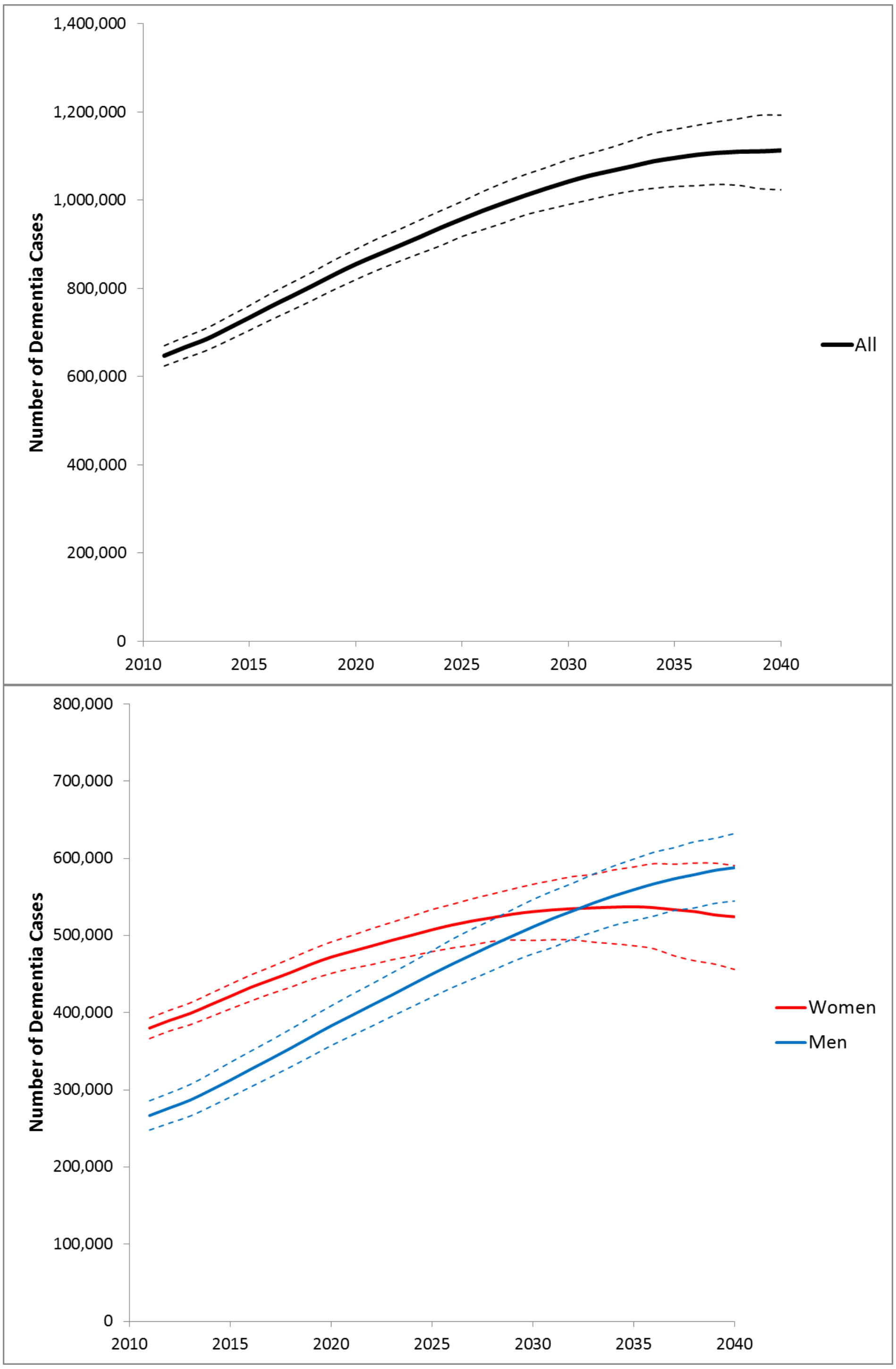

Dashed lines represent 95% uncertainty intervals.

Reference List

(1) Chertkow H, Nasreddine Z, Joanette Y, Drolet V, Kirk J, Massoud F et al. Mild cognitive impairment and cognitive impairment, no dementia: Part A, concept and diagnosis. *Alzheimers Dement* 2007; 3(4):266-282.

(2) Steptoe A, Breeze E, Banks J, Nazroo J. Cohort profile: the English longitudinal study of ageing. *Int J Epidemiol* 2013; 42(6):1640-1648.

(3) Jackson SE, van Jaarsveld CH, Beeken RJ, Gunter MJ, Steptoe A, Wardle J. Four-year stability of anthropometric and cardio-metabolic parameters in a prospective cohort of older adults. *Biomark Med* 2015; 9(2):109-122.

(4) Rogers NT, Marshall A, Roberts CH, Demakakos P, Steptoe A, Scholes S. Physical activity and trajectories of frailty among older adults: Evidence from the English Longitudinal Study of Ageing. *PLoS One* 2017; 12(2):e0170878.

(5) Demakakos P, Hamer M, Stamatakis E, Steptoe A. Low-intensity physical activity is associated with reduced risk of incident type 2 diabetes in older adults: evidence from the English Longitudinal Study of Ageing. *Diabetologia* 2010; 53(9):1877-1885.

(6) SOC2010 volume 3: the National Statistics Socio-economic classification (NS-SEC rebased on the SOC2010). 2017.

(7) Banks J, Lessof C, Nazroo J, Rogers N, Stafford M, Steptoe A. Financial circumstances, health and well-being of the older population in England. The 2008 English Longitudinal Study of Ageing (Wave 4). 2010. The Institute for Fiscal Studies.

(8) Craig R, Deverill C, Pickering K. Quality Control of Blood, Saliva and Urine Analytes. Health Survey for England 2004, Methodology and Documentation (Vol 2). The Information Centre, London, UK, 34-41 (2006). 2017.
